# Ultrafast Spectroscopies of Nitrophenols and Nitrophenolates in Solution: From Electronic Dynamics and Vibrational Structures to Photochemical and Environmental Implications

**DOI:** 10.3390/molecules28020601

**Published:** 2023-01-06

**Authors:** Sullivan Bailey-Darland, Taylor D. Krueger, Chong Fang

**Affiliations:** Department of Chemistry, Oregon State University, 153 Gilbert Hall, Corvallis, OR 97331, USA

**Keywords:** nitrophenol compounds, ultrafast spectroscopy, photophysics and photochemistry, charge transfer, molecular rotor, femtosecond stimulated Raman

## Abstract

Nitrophenols are a group of small organic molecules with significant environmental implications from the atmosphere to waterways. In this work, we investigate a series of nitrophenols and nitrophenolates, with the contrasting *ortho*-, *meta*-, and *para*-substituted nitro group to the phenolic hydroxy or phenolate oxygen site (2/3/4NP or NP^−^), implementing a suite of steady-state and time-resolved spectroscopic techniques that include UV/Visible spectroscopy, femtosecond transient absorption (fs-TA) spectroscopy with probe-dependent and global analysis, and femtosecond stimulated Raman spectroscopy (FSRS), aided by quantum calculations. The excitation-dependent (400 and 267 nm) electronic dynamics in water and methanol, for six protonated or deprotonated nitrophenol molecules (three regioisomers in each set), enable a systematic investigation of the excited-state dynamics of these functional “nanomachines” that can undergo nitro-group twisting (as a rotor), excited-state intramolecular or intermolecular proton transfer (donor–acceptor, ESIPT, or ESPT), solvation, and cooling (chromophore) events on molecular timescales. In particular, the *meta*-substituted compound 3NP or 3NP^−^ exhibits the strongest charge-transfer character with FSRS signatures (e.g., C–N peak frequency), and thus, does not favor nitroaromatic twist in the excited state, while the *ortho*-substituted compound 2NP can undergo ESIPT in water and likely generate nitrous acid (HONO) after 267 nm excitation. The delineated mechanistic insights into the nitro-substituent-location-, protonation-, solvent-, and excitation-wavelength-dependent effects on nitrophenols, in conjunction with the ultraviolet-light-induced degradation of 2NP in water, substantiates an appealing discovery loop to characterize and engineer functional molecules for environmental applications.

## 1. Introduction 

Nitrophenol molecules are compounds that have found themselves at the intersection of many fields of science, industry, and chemistry. They are of great interest in environmental chemistry [1,2,3], toxicology [4,5,6], atmospheric chemistry [7,8,9,10], chemical and bioengineering [2,11], computational chemistry [12,13,14], and ultrafast spectroscopy [15,16,17,18]. From an environmental perspective, nitrophenols are compact, prototypical examples and building blocks of nitroaromatic compounds, which are used for and produced through a wide variety of industrial applications and are emitted from diesel and gasoline engines. They are considered toxic or carcinogenic, and are linked to the formation of reactive oxygen species and oxidative stress [5]. In addition, *ortho*-nitrophenol (*o*-NP, or 2NP) is a source for the formation of nitrous acid (HONO) in the atmosphere and waterways [9,19,20], which then forms hydroxyl radicals (•OH). Notably, hydroxy radicals can be produced in a variety of environments and are considered powerful oxidants that can degrade organic compounds via oxidative stress reactions [21,22]. These radicals can promote the production of ozone (O_3_) and small particulate matter with a diameter of less than 2.5 µm (PM2.5), thus rendering nitroaromatic molecules as air pollutants, which can lead to smoggy air conditions, low air quality, and acid rain, especially in urban areas [18]. Computationally, the relative simplicity of the nitrophenols compared to larger molecules allows efficient and reliable modeling of key processes, especially the excited-state intramolecular proton transfer (ESIPT) reaction that is possible for *o*-NP [14,16].

In particular, the formation of nitrous acid from *o*-NP is found to be a result of photoexcitation [9,10,18], which has inspired ultrafast molecular spectroscopic studies of the behavior of *o*-NP in the excited state in order to characterize this intrinsically transient process. Meanwhile, systematic comparisons between a series of nitrophenols also serve as model systems to study the excited-state dynamics of larger, more complex aromatic molecules with both electron-donating and electron-withdrawing substituents at various positions of an aromatic ring system [16]. A closely related example is 3-nitrotyrosine, in which an electron-withdrawing nitro group at the *ortho* site to an electron-donating hydroxy group (or –O^−^ group) of the phenol ring allows the molecule to reach a twisted intramolecular charge-transfer (TICT) state and efficiently relax after photoexcitation [23].

There have been a variety of studies on both the photolysis and relaxation dynamics of *o*-NP in particular, using both experimental and computational methods. Studies on the gas-phase photolysis of *o*-NP have found that ultraviolet (UV) light ranging from 250 to 350 nm (across the UVC/200–280 nm, UVB/280–320 nm, and UVA/320–400 nm range) results in photolysis into HONO and OH radicals, through a mechanism involving proton transfer to the nitro group followed by the dissociation of HONO from the benzene ring [10,15]. Measurements of the ultrafast dynamics have been conducted both in the gas phase using photoelectron experiments and in solution using transient absorption spectroscopy, which revealed complex relaxation mechanisms involving ESIPT and efficient intersystem crossing (ISC) to triplet states [16,24,25]. It is worth noting that long-lived triplet states (>100 ps) have been observed in the gas phase, benzene, and n-hexane, but not in water. Quantum calculations of the excited-state trajectories agree with these results and suggest that a transition from the S_1_ to the T_2_ state is favored due to proximity to the Franck–Condon region and that hydrogen transfer likely occurs after subsequent internal conversion (IC) to the T_1_ state [14], while the nitro and carbonyl groups in the molecular framework aid the spin-orbit coupling (SOC) to enhance ISC [26,27].

However, there has been limited work to date on *meta*-nitrophenol (*m*-NP, or 3NP), *para*-nitrophenol (*p*-NP, or 4NP), and their deprotonated counterparts. Past work on *m*-NP and *p*-NP excited with 322 nm light has shown the existence of long-lasting absorption in chloroform, attributed to triplet state formation during relaxation [17]. Previous experiments on nitrophenolates demonstrate significant effects of the substituent placement on the excited-state relaxation dynamics, but have little evidence of triplet formation, thereby suggesting relaxation via IC to S_0_ rather than an ISC process to a triplet state [17]. Meanwhile, there is no previous work using a shorter excitation wavelength (e.g., in the UVC region below 280 nm) on nitrophenols or nitrophenolates in solution. While UVC light is generally higher-energy than that found in the environment due to the ozone layer of Earth’s atmosphere blocking UVC radiation from the sun [7], the dynamics in the condensed phase can reveal valuable insights into the higher-lying excited-state relaxation mechanisms available to nitrophenol molecules in solution and can be compared to dynamics observed in the gas phase with similar excitation energies across the UV range. Furthermore, these findings are likely relevant to relaxation mechanisms for other nitroaromatic compounds that can be excited to higher-lying excited states in the environment (especially under UVA and UVB light irradiation that reaches Earth’s surface) or under artificial light sources.

This investigation focuses on the excited-state dynamics of nitrophenolates and nitrophenols (which can be in equilibrium with each other in natural environments) in solution via femtosecond transient absorption (fs-TA) spectroscopy using higher-energy/UVC excitation (in contrast to visible light excitation) to track the molecular relaxation pathways, complemented by steady-state electronic measurements, ground-state femtosecond stimulated Raman spectroscopy (GS-FSRS), and quantum calculations. The comprehensive and correlated results indicate significant differences in the relaxation dynamics of nitrophenols and nitrophenolates when excited to higher-lying electronic states, and the prominent formation of charge-transfer (CT) and/or TICT states, as well as demonstrate key differences in excited-state evolution due to the protonation state, solvent, and substituent placement of a light-sensitive molecule (chromophore). Perhaps most interestingly, our investigations led to the discovery of previously unreported photolysis with clear macroscopic signatures of the protonated *o*-NP in aqueous solution, accompanied by a glimpse of its ultrafast dynamics among a series of contrasting molecular systems.

## 2. Results

### 2.1. Steady-State Electronic Spectroscopy of a Series of Nitrophenols and Nitrophenolates in Solution

**Figure 1:**
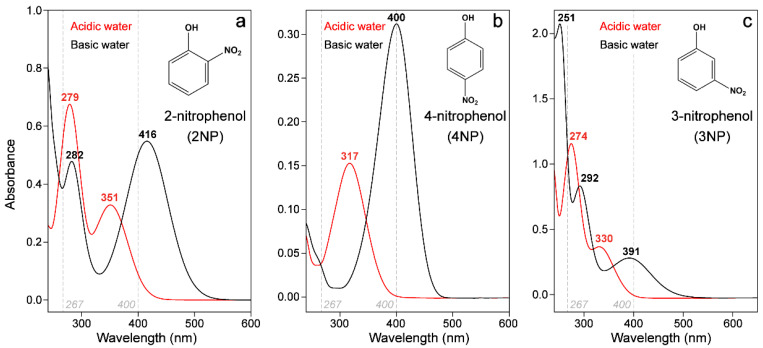
Steady-state absorption measurements of the three nitrophenols (red) and their deprotonated forms (black) in water. The wavelength of each prominent peak is indicated for (**a**) 2NP, (**b**) 4NP, and (**c**) 3NP in acidic (red) and basic (black) aqueous solutions. The pHs of the acidic and basic water solutions were ~4 and 10, respectively; exact values are in the Supplementary Materials (see Figure S1). The concentrations of each nitrophenol sample are identical across acidic and basic conditions to manifest the difference in absorptivity between the protonated and deprotonated molecules in aqueous solution. The two excitation wavelengths at 267 and 400 nm are denoted by vertical dashed gray lines.

Steady-state absorption profiles of the nitrophenols show clear peaks in the blue-to-UV region for all molecules, and clear differences between the protonated and deprotonated molecules. Absorption measurements taken at a variety of pHs in aqueous solutions experimentally validate the previously reported p*K*_a_ values for nitrophenols [2]: ~7.2, 8.4, and 7.1 for 2NP, 3NP, and 4NP, respectively (Figure S1). The changed p*K*_a_ of 3NP versus 2/4NP hint at the altered electronic effects of the *meta*- versus *ortho*-/*para*-substituent [12,28]. All the nitrophenolates have a strong peak around 400 nm in water, which is attributed to transitions with a significant CT character [17]; this result warrants further investigation via a structural technique such as GS-FSRS (see Section 2.8 below). The C–N stretching frequencies (at the “acceptor” site, –NO_2_) of the protonated versus deprotonated nitrophenols can be compared to evaluate the prominence of the CT state, with additional verification upon inspection of the nitrophenolate C=O stretching frequency (at the “donor” site) to further validate the CT state. Previous calculations attribute this transition to S_0_→S_1_ (with ππ* character) for 2NP^−^ and 4NP^−^, but the assignment remains unclear for 3NP^−^. Calculations predict an S_0_–S_1_ transition around 500 nm, while previous spectra taken in basic acetonitrile (with sodium *tert*-butoxide as a base) and gas phase show a ~475 nm peak [12]. However, due to the lack of a lower-energy electronic absorption peak, we can tentatively attribute the 391 nm peak (Figure 1c) to an S_0_–S_1_ transition of 3NP^−^, in accord with its proximity to the corresponding peak wavelengths in 2NP^−^ and 4NP^−^ (Figure 1a,b). The control measurements of 3NP^−^ in acetonitrile that we performed (with an organic base of 1,8-diazabicyclo [5.4.0]undec-7-ene or DBU) showed that the absorption peak red-shifts significantly (from ~391 to 432 nm; see Figure S1 appendix figure), supporting the assignment. Future studies, particularly on the solvatochromic shift of this electronic transition [29,30], may yield additional insights into the interplay between the *meta*-substituent and solvent molecules.

These results are consistent with the calculated natural orbitals that tend to localize the highest occupied molecular orbital (HOMO) more on the phenolate and the lowest unoccupied molecular orbital (LUMO) on the nitro group, resulting in varying CT characteristics dependent on the substituent placement [12]. The excitation of 3NP^−^ displays the most significant CT since the ground-state (S_0_) wavefunction places little electron density on the nitro group, while prominent electron density is placed on the nitro group in the excited state, thus leading to the decoupled donor HOMO and acceptor LUMO regions, and a much-reduced transition oscillator strength. In contrast, the HOMO and LUMO have much more overlap in 2NP^−^ and 4NP^−^ due to the quinoid resonance structures possible for these molecules [12]. There has been less investigation into the higher-energy spectral region, and some preliminary calculations for 2NP^−^ found that the next set of transitions correspond to S_0_–S_5_ and S_0_–S_6_ (predicted in water at 249 and 237 nm, using the second-order approximate coupled-cluster CC2 method), inspiring future computational investigations.

The absorption peaks of the protonated nitrophenols are significantly blue-shifted from their deprotonated forms in all molecules, and the energetic differences between the two forms can be calculated from the experimental peaks to be 0.55, 0.59, and 0.81 eV for 2NP, 3NP, and 4NP, respectively. Past calculations of 2NP attributed the reddest peak (351 nm) to the S_0_–S_1_ transition and the next peak (279 nm) to the S_0_–S_4_ transition, both of which show the same CT process from the phenol to the nitro group [16]. Future computational work could be performed, particularly on 3NP and 4NP, in order to determine the exact nature of the higher-energy transitions for the protonated molecules with solvent dependence.

### 2.2. Ultrafast Electronic Spectroscopy of Nitrophenolates in Water

**Figure 2:**
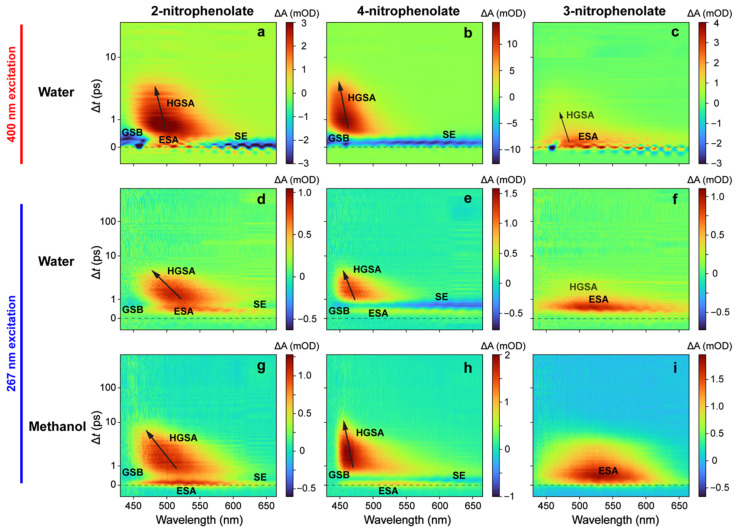
Semilogarithmic contour plots of fs-TA spectra of various nitrophenolates in water and methanol. The logarithmic scaling is for time points after 1 ps. The 400 nm excitation data for (**a**) 2NP^−^, (**b**) 4NP^−^, and (**c**) 3NP^−^ are compared with the 267 nm excitation data for (**d**) 2NP^−^, (**e**) 4NP^−^, and (**f**) 3NP^−^ in water, as well as the 267 nm excitation data for (**g**) 2NP^−^, (**h**) 4NP^−^, and (**i**) 3NP^−^ in methanol, respectively. Longer time windows are shown for panels (**d**–**i**) to expose the weak yet discernible positive signal after a ~100 ps time delay for nitrophenolates after 267 nm excitation compared to 400 nm excitation (panels (**a**–**c**)). For the methanol data, the spectra shown have an average of five pre-excitation spectra subtracted to remove the nonzero background (see Section 2.3 below for details). Major TA bands are labeled in each panel, with prominent spectral shifts indicated by the tilted black arrows. Time zero of photoexcitation is denoted by the horizontal dashed line in each panel.

We performed femtosecond transient absorption (fs-TA) measurements of three nitrophenolates (NP^−^) in basic water upon 400 nm excitation following previous experiments conducted by Michenfelder et al., under similar conditions [17], which serve as a fitting comparison to the new series of 267 nm excitation experiments in this work. 4NP^−^ has a broad initial stimulated emission (SE) feature, followed by a weak excited-state absorption (ESA) feature that evolves into a hot ground-state absorption (HGSA) band, which then blue-shifts as it decays (Figure 2b). Given that a similar TA spectral pattern was observed and reported by us for nitrotyrosine (also with a rotatable nitro group on the phenolate ring) upon 400 nm excitation, the SE band likely originates from a TICT state that forms on the ~0.2 ps timescale (Table 1) and is limited by the cross-correlation time of the experimental optical setup [23]. Importantly, a TICT state is known to have red-shifted SE wavelengths versus steady-state fluorescence peaks, lending support to this assignment [31,32,33,34]. The dynamics are similar in 2NP^−^ (Figure 2a), except that the initial feature has contributions from both SE (at wavelengths greater than 550 nm) and ESA (centered around 500 nm). The spectral features and lifetimes, derived from global analysis (see Table 1 below and Figure S2), are consistent with previous results [17]. In sharp contrast, under the same experimental conditions, 3NP^−^ exhibits no SE features, and is thereby replaced by a broad initial ESA band that quickly decays and blue-shifts, followed by a weak HGSA feature (Figure 2c). The absence of an SE band suggests that the aforementioned TICT state may not be accessible in 3NP^−^ (see detailed analysis with computational evidence below in Section 2.4). From global analysis of the fs-TA spectra, the ESA band lifetime and the absorption profile of 3NP^−^ are almost identical to past measurements [17], but an additional feature assigned to HGSA was also identified (Figure S2c). This HGSA band is much less intense than that in 2NP^−^ and 4NP^−^ (Figure 2a–c), but with similar lifetimes and absorption profiles (Figure S2a–c, green traces), which is consistent with the diminished effect of nitro-group twisting on the potential energy surface (PES) of 3NP^−^ (see Section 2.4 below). The absence of major changes to the 3NP^−^ PES upon nitro-group twisting leads to a weaker HGSA feature that displays a reduced blueshift, because the displacement along the reaction coordinate from the excited to the ground state is inherently smaller for 3NP^−^ when compared to the other nitrophenolates. Notably, the weak HGSA feature of 3NP^−^ in water and the lack of an initial SE band do not contradict the previous analysis: SE was only observed at wavelengths greater than 640 nm in chloroform (there is no clear SE band in the visible region—only a weak SE signal at 950 nm in water) [17], and HGSA is expected to exist after the prompt nonradiative relaxation into S_0_.

**Figure 3:**
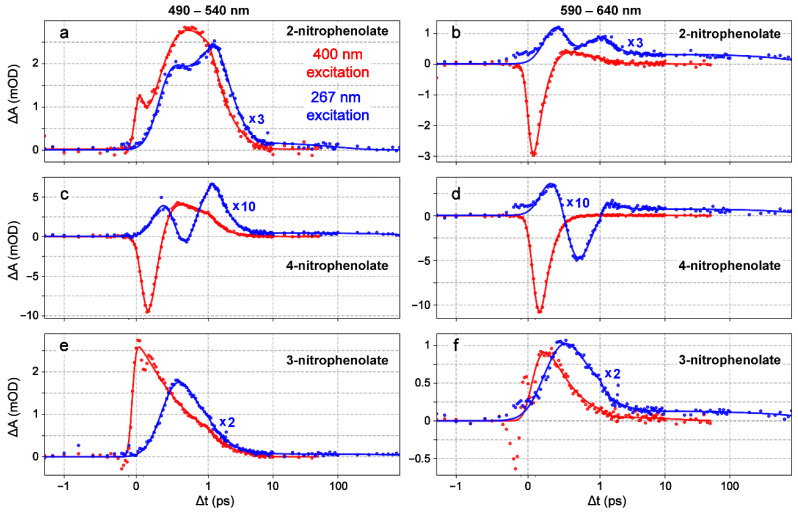
Fs-TA spectra of nitrophenolates in water at two different probe-wavelength ranges under 400 nm (red) or 267 nm (blue) excitation. Spectral data (filled dots) are averaged over the ranges of (left) 490–540 nm and (right) 590–640 nm for (**a**,**b**) 2NP^−^, (**c**,**d**) 4NP^−^, and (**e**,**f**) 3NP^−^, respectively, and overlaid with the best fits (color-coded solid lines) from the probe-dependent analysis (see Section 4.5 for detailed methods).

**Figure 4:**
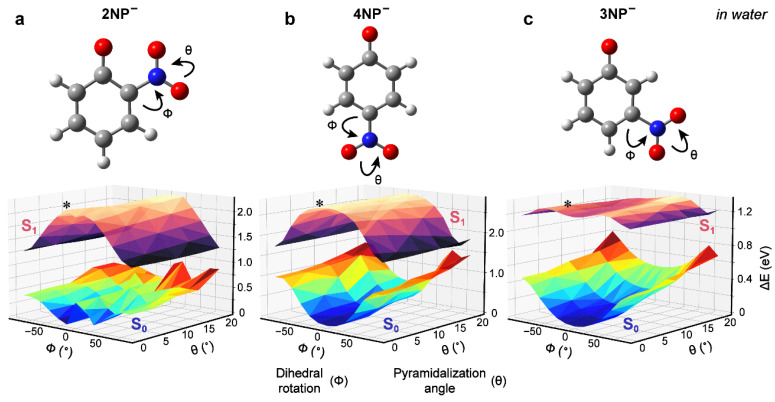
2D-coordinate scans of S_1_ and S_0_ energies for the nitrophenolates along the nitroaromatic dihedral angle and pyramidalization angle in water. The two types of nuclear motion are depicted by curved arrows for (**a**) 2NP^−^, (**b**) 4NP^−^, and (**c**) 3NP^−^, with the potential energy surfaces (PESs) depicted below each chemical structure. The current figure perspectives are selected to illustrate the calculated multidimensional PESs (essentially displaying a 3D picture in 2D space) and focus on the comparative similarities and differences between three regioisomers of nitrophenolates in water. Details of the calculation are shown in the Methods section below. At each point, all other coordinates were relaxed in the S_1_ state according to the TD-DFT optimization using the B3LYP level of theory, 6-31G+(d,p) basis sets, and the IEFPCM water solvent (default method for implicit solvent in Gaussian software [44]). Energies are shown in electron volts (eV) relative to the lowest-energy S_0_ geometry. The Franck–Condon region accessed by vertical excitation from the optimized ground-state geometry is denoted by an asterisk (*) in each panel.

While 400 nm excitation leads to an immediate SE feature for both molecules (see Figure 2a,b, and red traces in Figure 3a–d), the 267 nm excitation shows an initial broad ESA feature before any other features appear in the TA dynamics of 2NP^−^ (Figure 2d) and 4NP^−^ (Figure 2e); this can be better seen in the probe-dependent plots (see blue traces in Figure 3a–d) and also explains the ground-state bleaching (GSB) buildup with an apparent temporal delay on the sub-ps timescale. This point is further supported by the global analysis results in Figure S2d,e, which show the retrieved initial positive ESA band (black) strongly overlapping with a negative band (red; see below for details). After the initial positive feature, the TA dynamics resemble those after 400 nm excitation. Both 2NP^−^ and 4NP^−^ exhibit a broad dip in absorption due to the ultrafast rise in an SE feature (<0.1 ps rise time constant from the fitting, Table 2) that is overlapped with positive ESA features before and after it (Figure 3a–d, blue traces), displaying differences in the relative intensity compared to the 400 nm excitation cases (Figure 3a–d, red traces). Subsequently, there is a broad positive absorption band which narrows and blue-shifts. The global analysis reveals two underlying components responsible for this feature: a ~0.5 ps largely absorptive feature with a negative peak around 460 nm that could be associated with GSB, followed by a blue-shifted positive feature with a ~1.3 ps lifetime (see Figure S2d,e for details). The first feature can be attributed primarily to an ESA (with clear contributions from the overlapping GSB and SE bands), while the second blue-shifted feature represents HGSA. In addition, the HGSA peak is initially red-shifted compared to that after 400 nm excitation, which is likely due to an altered excited-state relaxation pathway that reduces the S_0_–S_1_ energy gap after 267 nm excitation (see Figure 2d,e versus Figure 2a,b); however, the associated HGSA band decay time constants are largely conserved across 400 and 267 nm excitations (see Table 2 and Figure S2) due to the same solvent (water) having a typical solvation time of ~1 ps and likely facilitating the HGSA relaxation in S_0_ [35,36].

The spectrum for 3NP^−^ after 267 nm excitation shows a prolonged broad absorption band (Figure 2f and Figure 3e,f) that is redder and longer-lasting than that after 400 nm excitation. This observation hints that a different excited state could be accessed after 267 nm excitation, and the delayed rise in a prominent ESA band on the sub-ps timescale (blue traces in Figure 3e,f) originates from an S_1_′ state that differs from the S_1_ state immediately accessed by 400 nm light [37]. The global analysis yields a ~0.6 ps lifetime component with no clear peak shift, which is reminiscent of the ESA band after 400 nm excitation (~0.5 ps lifetime). Previous experiments on 3NP^−^ suggest that the molecule remains in the excited state for longer than other nitrophenolates due to its higher CT character in the excited state, which can undergo charge recombination on longer timescales than the nitro-group twisting in 2/4NP^−^ [17]; this is also consistent with the proposed lack of a prominent nitro-twisting coordinate on the energetics of 3NP^−^ (see below). No clear HGSA is found in the global analysis (Figure S2f), but it is faintly visible in the 2D-contour data plot (Figure 2f); this is likely due to low signal strength (with less light-induced nuclear coordinate change between the excited state and ground state), as well as being obscured by the strong preceding ESA band of 3NP^−^ in water.

One of the most interesting differences is the presence of long-lasting absorption in all three nitrophenolates after 267 nm excitation. Global analysis fits the feature with a lifetime over 1 ns for all three nitrophenolates in water (see Table 1 and Figure S2). This feature can be seen primarily at wavelengths greater than 550 nm and also in the redder-region probe-dependent fits (see Figure 3b,d,f and Table 2). This unexpected result is important since dynamics beyond 100 ps have not been observed or reported for nitrophenolates [17]. However, a long-lasting absorption band was observed in the protonated nitrophenols in organic solvents after 400 nm excitation and in gas-phase experiments on 2NP [16,25]. In those cases, the absorption band was attributed to a triplet state after ISC. Moreover, previous experiments and calculations suggest that nitrophenol triplet state lifetimes are surprisingly short, in the order of hundreds of ps to ns; this is likely due to efficient ISC pathways back to the ground state [14,24], and is consistent with an increasing $n\pi^{*}$ character upon nitro twisting in protonated chromophores and the increased access to the triplet manifold [26,38]. Our quantum calculations, conducted by scanning two nuclear coordinates (see Section 2.4 and Figure 4 below) in the excited state of nitrophenolates, show that nitro-group twisting can lead to an energy approach between the S_1_/T_1_ states [14], providing further evidence that this mechanism may increase ISC probability. Moreover, this evidence could rationalize the aforementioned “surprisingly short” triplet state lifetimes, because the triplet state quickly reaches a conical intersection (CI) via facile nitro twists to efficiently deactivate the excited state. Accordingly, the long-lived “trapped state” observed in this work (see Figure 2 and Figure 3) could originate from a higher-lying triplet state absorption feature, which is consistent with broad and weak absorption bands as the spectral signatures of triplet states [39,40,41]. Since there is no high-level computational work about ISC in solvated nitrophenolates, the assignment of this long-lived band remains tentative. In addition, these long-lived absorption peaks observed in our experiments are much redder (around 615 nm) than the absorption peaks (around 500 nm) reported for triplet states in nitrophenols [16,17], substantiating the altered electronic structures between nitrophenols and nitrophenolates due to the change in protonation states (e.g., generally speaking, bluer electronic absorption peaks of nitrophenols than nitrophenolates; see Figure 1).

Moreover, it is worth noting that there is a faint early red positive feature that appears upon 267 nm excitation, followed by the much more prominent broad ESA. This initial feature can be better seen in the probe-dependent fits around 600 nm centered at time zero (see blue dots in Figure 3b,d,f), but is not present at lower probe-wavelength regions (Figure 3a,c,e). Fitting this faint feature using global analysis was not feasible due to its low intensity and short lifetime within the cross-correlation; instead, our fitting procedures reported a delayed time zero of ~420, 500, and 370 fs for 2NP^−^, 4NP^−^, and 3NP^−^, respectively. This feature was fitted via the probe-dependent fitting algorithm using several ultrafast rise and decay components around time zero, but to focus on a minimal number of temporal components with physical insights, the initial weak ESA feature was not fitted (Figure 3; see above), but instead, approximated with a relatively large FWHM representing the apparent pump–probe pulse overlap (Table 2). While the instrument response time of our current optical setup (see Section 4.3 below for experimental methods) was relatively broad (cross-correlation of ~200–300 fs with 267 nm excitation) and the true time zero may be slightly offset from the calibrated time zero, it is unlikely there is a delayed time zero beyond 500 fs in a carefully constructed experiment. Importantly, other spectra collected on the same day (see Figure S3, catechol in water) with the same experimental setup did not show this delay, suggesting that the spectral origin is the intrinsic molecular dynamics of photoexcited nitrophenolates in water. In particular, the red-shifted small ESA band likely arises from S_n_ (n ≥ 2) with a <300 fs lifetime [32,37], and the subsequent prominent ESA band arises from the delayed arrival of a lower-lying S_1_′ state (with the prime denoting a different state from the S_1_ state that is directly accessible by 400 nm excitation) out of the initially excited S_n_ state, all on the sub-ps timescale.

### 2.3. Ultrafast Electronic Spectroscopy of Nitrophenolates in Methanol

Nitrophenolates in methanol (Figure 2g,h,i) exhibit qualitatively similar dynamics to those in water (Figure 2d,e,f) after 267 nm excitation, which can be readily visualized by the 2D-contour plots of fs-TA spectra for 2NP^−^, 4NP^−^, and 3NP^−^, respectively. Moreover, global analysis yields similar spectral components in the same order in both solvents (Figure S2d–i). For 2NP^−^ and 4NP^−^, a strong initial ESA band is followed by an intensity drop due to overlap with a negative SE band; then, a clear HGSA band emerges (Figure 2g,h). From the global analysis, the excited-state lifetimes remain mostly unchanged, while the HGS dynamics are lengthened much more significantly versus their counterparts in water (Table 2), which is reflective of a longer average solvation time (~5 ps) of methanol [35,42,43]. The observed SE band is also much weaker in methanol, particularly for 2NP^−^ (barely visible in Figure 2g), which indicates that the less polar and bulkier methanol cannot accommodate the CT/TICT state as effectively as water. The difference in HGS lifetimes can be attributed to reduced solute–solvent interactions in methanol due to its lower polarity and larger size than water; this is more evident due to the longer relaxation times in S_0_ than S_1_ of the chromophore because the excited state decays to HGS before appreciable solvation of the chromophore can occur.

For 3NP^−^, there are more substantial differences between the TA dynamics in methanol (Figure 2i) and water (Figure 2f) upon 267 nm excitation, which is consistent with the aforementioned stronger CT character of 3NP^−^ than 2/4NP^−^ in gas phase and solution [12,17]. The ESA band has a much longer lifetime in methanol and can be fitted by two components in global analysis: a broad absorption band with a ~0.2 ps lifetime, followed by a feature almost identical to the ESA seen in water (Figure S2f) but with a 1.3 ps lifetime (Figure S2i). Both features could be present in water as well, but their reduced lifetimes prevent precise deconvolution at early times (see the black trace with a ~0.6 ps lifetime in Figure S2f). The significant lengthening of the excited-state features in methanol matches previous results, where the excited-state lifetime of 3NP^−^ clearly increases in organic solvents (e.g., after 400 nm excitation in chloroform) [17]. The increase is much more dramatic in previous results (a factor-of-10 increase in lifetimes), likely owing to the more substantial polarity difference between water and chloroform (1 vs. 0.26, while the relative polarity value is 0.76 for methanol) and the lack of hydrogen bonding in chloroform. In other words, less polar solvents can less readily accommodate the CT state and facilitate CT recombination than more polar (e.g., water) solvents, while increased solvent–solute interactions can quench the excited state more in polar solvents [32]. There is also an observable HGSA feature in methanol, retrieved from global analysis with a 5.7 ps lifetime (see Table 1 and Figure S2i), lengthened from the HGSA lifetime in water (Figure S2a–e). This assignment is based on the discernible narrowing of the positive band, the profile similarity to the 400 nm HGSA feature, the lifetime similarity to the HGS decay for 2NP^−^ and 4NP^−^ in methanol, and the peak blueshift of the positive feature as it decays (Figure 2i).

There are also two common differences in the spectra for all nitrophenolates in methanol compared to water. While there is a delayed onset of substantial features (i.e., a faint early absorption band) in water, there is no such delay in methanol, which can clearly be seen in the probe-dependent data comparisons between chromophore samples in the two solvents (Figure S4). Since 267 nm excitation likely brings the nitrophenolates to a similar electronic state (e.g., S_n_) in both solvents, the nascent ESA features associated with the lower-lying S_1_′ state (S_1_ state is reserved for 400 nm excitation; see above and more discussions below) emerge faster in methanol due to faster S_n_→S_1_′ relaxation. The second difference is the lack of a clear long-lasting absorption band in methanol (see green traces in Figure S4). Notably, the TA data in methanol (for all six measured samples: 2/3/4NP and NP^−^) show a broad positive absorption band at the start of the measurement window (~2 ps before the pump pulse) and toward the end (~900 ps after the pump pulse) (see Figure S5 for details). This absorption band can thus be explained by a long-lived trapped state signal, but for the absorption to appear before the pump pulse, it would need to have a lifetime in the order of milliseconds (given the laser repetition rate of 1 kHz; see Section 4.3 below). The subtraction of this feature (by subtracting the TA data for the first five time points) makes analysis of any long-lasting absorption challenging and less reliable, though it enables us to obtain more accurate lifetimes and spectral dynamics on the fs-to-ns timescale and provides deep insights into the initial electronic dynamics of these nitrophenolates in two contrasting solvents.

### 2.4. Excited-State Quantum Calculations of Nitrophenolates in Water

To provide further insights into nitrophenolate dynamics, we performed quantum calculations of the S_0_ and S_1_ energies for the three nitrophenolates in water that reveal clear differences in the preferred molecular configurations. At each step, the excited-state structure was optimized using the time-dependent density functional theory (TD-DFT) method with the B3LYP level of theory and 6-31G+(d,p) basis sets, and the corresponding S_0_ energy at each optimized structure was obtained from the Gaussian output file (e.g., using the S_1_ state energy minus the emission peak energy) [44]. Scanning the dihedral angle of the nitro group (measuring nitro-group twisting/rotation with respect to the aromatic ring) and the pyramidalization angle (measuring the folding of the oxygens toward each other) shows a clear path toward a CI for 2NP^−^: starting from the planar configuration (preferably in the ground state at thermal equilibrium, with both the nitro dihedral and pyramidalization angles at zero degrees), the nitro-group rotation and pyramidalization both raise the S_0_ energy and lower the S_1_ energy (Figure 4a), which is characteristic of nuclear motions that could lead to a CI. This finding suggests that the relaxation mechanism is not as simple as pure nitro rotation, and the interaction between the phenolate oxygen and the nitro group in the excited state leads to a multidimensional potential energy landscape, which is consistent with the aforementioned representative two-coordinate scan to approach a CI. Regardless, these results align with previous calculations on the protonated 2NP, where nitro-group rotation and proton transfer facilitate a CI [16]. Note that we performed these readily accessible and economical calculations to simulate the ground- and excited-state energy trend as a function of two representative internal angles [23,32,36], and it was not intended to quantitatively reproduce the experimental values. In general, the S_1_–S_0_ energy gaps illustrated in Figure 4a,b are smaller than the experimental SE emission wavelengths for 2/4NP^−^, while there is a lack of an initial SE band for 3NP^−^ (seen only at ~950 nm; see Section 2.2 above). Moreover, the experimental SE band follows the trend: 4NP^−^ (bluest; see Figure S2b,e), 2NP^−^ (intermediate; see Figure S2a,d), and 3NP^−^ (reddest), which is consistent with the calculated trend for the S_1_–S_0_ energy gaps of these nitrophenolates (see Section 4.4 for computational methods).

Similarly, 4NP^−^ clearly favors a rotated nitro group in the S_1_ state, also bringing the S_1_ and S_0_ energies closer to each other (Figure 4b). However, pyramidalization does not alter the energies of the states as much as for 2NP^−^ because the nitro group and the phenolate oxygen are not adjacent in 4NP^−^. From this calculation, 4NP^−^ likely undergoes predominantly nitro-twisting motions, with much less pyramidalization during relaxation than in 2NP^−^.

Interestingly, for 3NP^−^, the nitro-group twist is not a prominent factor along the reaction coordinate for excited-state energy dissipation (Figure 4c). This finding indicates that the electronic rearrangement and charge recombination likely dominate the excited-state decay over nuclear coordinate change; this is in accord with the enhanced CT character in 3NP^−^ (see Section 2.1 above and Section 2.8 below) that leads to a stronger C–N bond, thus making nitro-group rotation less feasible. Therefore, unlike in 2/4NP^−^, there is no clear nuclear motion involving the nitro group that allows the S_0_ and S_1_ states of 3NP^−^ to effectively approach (getting closer) in energy. Previous models for 3NP^−^ relaxation assumed nitro-group twisting to allow an efficient relaxation pathway back to S_0_ [17], whereas our further scans of the dihedral angle using a higher level of theory (6-311G++(d,p)) confirmed that the nitro twist (φ angle in Figure 4c) has a much smaller influence on the S_1_ PES of 3NP^−^ than 2/4NP^−^. Our work suggests that the elongated lifetime of the ESA band in 3NP^−^ (see Table 1) may be, at least in part, due to the difference in the nuclear motions of the nitro group in S_1_, which is likely in association with the smaller-scale and less facile nitro-group twisting motions due to a stronger C–N bond (with FSRS evidence presented below in Section 2.8). Further computational investigations of 3NP^−^, and the *meta*-substituted donor–acceptor systems in general, are needed to fully understand the effects of interplay between electronic and steric factors on the excited-state relaxation mechanisms of nitrophenolates and other related derivatives.

### 2.5. Potential Energy Surfaces (PESs) of Nitrophenolates in Water after Photoexcitation

**Figure 5:**
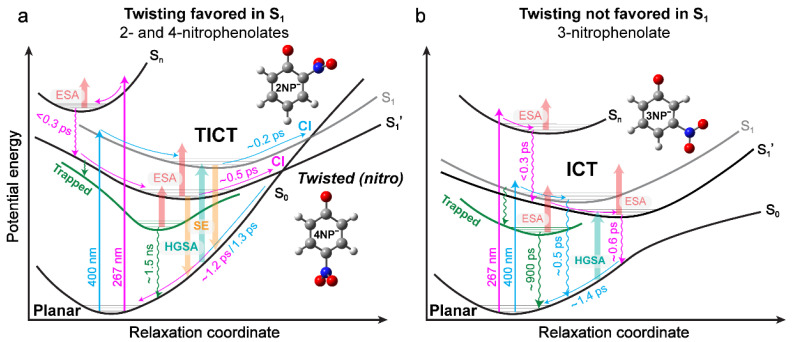
Schematics of the excited-state relaxation mechanisms of nitrophenolates in water. For (**a**) 2NP^−^ and 4NP^−^, nitro-group twisting enables a TICT state that leads to a sloped S_1_/S_0_ CI, whereas for (**b**) 3NP^−^, it does not twist much, and hence, accesses only an intramolecular charge transfer (ICT) state with no CI. Time constants for the pertinent processes are generally retrieved from global analysis of fs-TA spectra. The energy and relaxation coordinates are not scaled, while the colors for various processes roughly correspond to their associated wavelengths. The S_1_ state is reached via 400 nm excitation, which is different from the S_1_′ state reached via 267 nm excitation after the initial relaxation from S_n_. Representative ESA, SE, and HGSA features visible in the fs-TA data are indicated by “broad” maroon, orange, and green arrows, respectively.

To conclude the discussion on nitrophenolates, we note that similar dynamics can be predicted by similar electronic effects that are dependent on the substituent placement/location. When the substituents are *ortho* or *para* to each other (in 2NP^−^ or 4NP^−^), we generally observe the same electronic features after excitation. In contrast, when the substituents are *meta* to each other (in 3NP^−^), the dynamics change significantly. Interestingly, the steady-state macroscopic p*K*_a_ measurements yield the same conclusion: 3NP is different (Figure S1). Combining these measured differences and the results of the quantum calculations using Gaussian software [44] with respect to the nitro-group motions, we can model light-induced nitrophenolate relaxation in water as follows (Figure 5). With a 400 nm light, the *ortho*- or *para*-substituted molecules are vertically excited into S_1_, and rapidly slide down the PES to reach a TICT state that allows efficient relaxation back to S_0_ [23,32]. In particular, the light-induced efficient CT process (out of the Franck–Condon region [36]) and subsequent nitro-group twisting allow the molecule to quickly (<200 fs after excitation) reach a lower-lying TICT state with a significantly red-shifted SE band compared to the steady-state S_0_–S_1_ transition energy gap [23,45]. From this state, the molecule can undergo further small-scale structural motions to reach a sloped S_1_/S_0_ CI [23,32], and then, HGS relaxation over ~1–2 ps (aided by solvation since it is consistent with the longitudinal relaxation time of water [32,35,46]), resulting in a blue-shifting HGSA band observed in the 2D-contour plots of fs-TA spectra (Figure 2a,b).

For comparison, after the higher-energy 267 nm excitation, the nitrophenolate molecule is vertically excited to a higher-lying electronic state, S_n_ (likely S_5_ or S_6_ for 2NP^−^). This state could be responsible for the very faint ESA feature observed in long-wavelength regions of 2NP^−^ and 4NP^−^ (Figure 3b,d). To explain the excitation-dependent spectral differences, the excess photoexcitation energy likely guides the initial relaxation from S_n_ to a distinct S_1_′ state [37]. Overall, the S_1_′ state has several shared properties with the S_1_ state, in accordance with the generally similar features observed after both 267 and 400 nm excitations (Figure 2a–f). However, the following differences still show up. First, the S_1_′ state leads to a broad ESA feature before the appearance of any SE features, in contrast to the 400 nm excitation cases (e.g., see Figure 2d,e vs. Figure 2a,b). This observation signifies that the TICT state is promptly formed upon 400 nm excitation compared to the delayed arrival following 267 nm excitation. Second, since the molecule is unlikely to remain in the initially accessed S_n_ state for ~400 fs or more due to the steep slope out of the Franck–Condon region [32,36,37], the prominent ESA band (clearly seen in Figure 3b,d) arises from the rapidly accessed S_1_′ state after 267 nm light excitation (Figure 5a).

Relaxation from the TICT state (with the prominent, red-shifted SE feature) to the S_1_/S_0_ CI is also extended in S_1_′, from ~0.2 ps (400 nm excitation) to ~0.5 ps (267 nm excitation; see Table 1 and Figure S2) for 2/4NP^−^, which is characterized by slower decay of the SE feature after 267 nm excitation (Figure 3a–d). Calculations and previous models suggest that there is no clear energy barrier en route to an S_1_/S_0_ CI (e.g., Figure 4a,b); however, our TA data indicate that the molecule remains in the excited state for some time due to a sloped CI [17,23,32]; otherwise, a well-defined red-shifted SE band (but with no peak shift in the excited state) would not be present for 2NP^−^ (Figure 2a,d) and 4NP^−^ (Figure 2b,e) in water. This model matches the relaxation pathway of 3-nitrotyrosine (3NY), which is structurally identical to 2NP, apart from a substituent *para* to the phenolic OH group, wherein relaxation is also dominated by nitro-group twisting in the excited state. TA signatures and quantum calculations for the anionic 3NY manifest high similarities to 2NP^−^, while less spectral overlap and higher signal allowed a sloped CI to be identified less ambiguously [23]. Since the apparent HGSA band is redder after 267 nm excitation compared to 400 nm excitation, the S_0_-to-S_1_ energy gap reduction and the aforementioned lengthening of excited-state relaxation time imply extra relaxation in S_1_′ compared to S_1_ prior to passage through the CI. Moreover, since the HGS decay constant is largely conserved after both excitations, they likely access similar ground-state PESs (see the common S_0_ state in Figure 5a).

Notably, the S_1_′ state allows access to a long-lived trapped state, which is responsible for the TA signal that is redder than ~550 nm with a 1.3–1.6 ns lifetime for 2/4NP^−^ after 267 nm excitation (Figure S2d,e). The exact nature of this state cannot be determined from the TA data, with a limited signal-to-noise ratio and detection time window of ~1 ns, but previous experiments and calculations on protonated 2NP suggest some triplet state formation [24], which is consistent with higher-level excitations that promote ISC [38,47,48]. Due to models for 2NP which suggest bifurcation to a triplet state from S_1_, the trapped state could be reached from S_1_′. However, it is also possible that the trapped state is not a triplet state and/or the bifurcation occurs out of S_n_. Resolving these questions could inspire future experimental and computational studies.

The *meta*-substituted molecule (3NP^−^) exhibits a different relaxation process, owing to the electronic effects of the substituent placement (experimental evidence in Section 2.1 and Section 2.7, and Figure S1). Excitation at 400 nm directly accesses S_1_ (see Section 2.1) and, almost instantaneously, a CT state [32,36,49], which is responsible for the broad ESA feature (Figure 2c). Instead of relaxing into a TICT state, our calculations suggest that the molecule does not favor nitro-group twisting in S_1_; this is well supported by TA data where 3NP^−^ exhibits few spectral changes in the excited state (Figure 2c) compared to 2NP^−^ and 4NP^−^ with clearly evolving ESA and SE features (Figure 2a,b). The longer ESA lifetime of 3NP^−^ (~0.5 ps vs. 0.2 ps for 2/4NP^−^) hints that 3NP^−^ cannot reach an S_1_/S_0_ CI to efficiently return to S_0_. In addition, the weak HGSA features with little blueshift are perhaps our strongest evidence that the nitro group does not project strongly onto the reaction coordinate (see Figure 4c), and the photoexcited 3NP^−^ undergoes nonradiative internal conversion to the electronic ground state with much-reduced HGSA transition oscillator strength.

After exposure to 267 nm light, 3NP^−^ is excited to a higher-lying electronic state, again labeled as S_n_. Reminiscent of 2NP^−^ and 4NP^−^, an S_n_→S_n+1_ transition may be responsible for the faint early ESA feature around time zero (Figure 3f). The molecule then relaxes into an S_1_′ state (Figure 2f), similar to the S_1_ state reached after 400 nm excitation (Figure 2c with a similar ESA band). The pertinent excited-state relaxation mainly consists of CT recombination (i.e., electronic redistribution) and other subtle nuclear motions with a redder ESA band (than 2/4NP^−^), suggesting a smaller energy gap to some higher-energy state. The weak HGSA is also redder than that with 400 nm excitation (a similar pattern to 2/4NP^−^), again suggesting further relaxation in S_1_′ compared to S_1_. The most significant difference, similar to 2/4NP^−^, is that 267 nm light irradiation can access a trapped state of 3NP^−^ with a redder absorption band at later times (Figure 3f), which may be a triplet state and accessible from S_1_′ (Figure 5b) or S_n_.

The effect of methanol on all nitrophenolates is generally the same: it prolongs the HGSA lifetime with minimal influence on the excited state. There are weaker solvent–solute interactions between nitrophenolates and methanol than water, due to less hydrogen bonding and the lower polarity of methanol as a solvent; thus, the prominent CT state is not stabilized as effectively in methanol, which also lengthens the timescale for CT recombination. The most significant effect is on 3NP^−^, where the excited-state lifetime is greatly lengthened. In contrast to the 2/4NP^−^ relaxation via both ICT and nitro-group twisting (through an S_1_/S_0_ CI), 3NP^−^ relaxes via charge recombination after ICT (Figure 4c), so the solvent polarity can exert a larger effect on 3NP^−^ relaxation through interactions with the ICT state (Figure 5b). This interpretation is corroborated by previous results on the lifetimes of 3NP^−^ after 400 nm excitation in highly nonpolar solvents, where a more significant extension of the excited-state lifetime occurs [17].

### 2.6. Fs-TA Spectroscopy of Nitrophenols in Water after 267 nm Excitation

**Figure 6:**
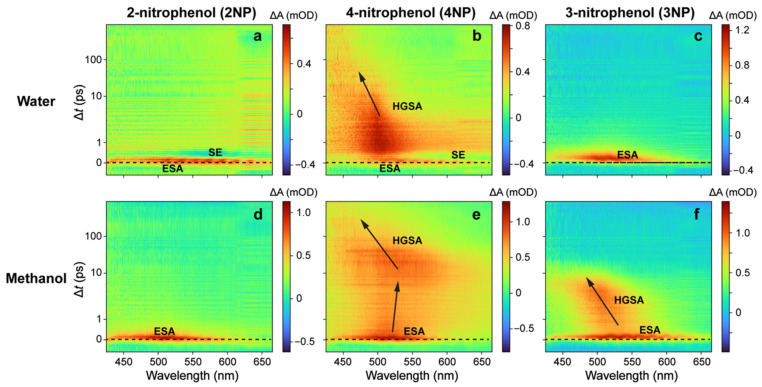
Semilogarithmic contour plots of fs-TA spectra of nitrophenols in water and methanol up to 900 ps after 267 nm excitation. The logarithmic scaling is for time points after 1 ps. The spectra of (**a**) 2NP, (**b**) 4NP, and (**c**) 3NP in water are compared with the spectra of (**d**) 2NP, (**e**) 4NP, and (**f**) 3NP in methanol. Key spectral features are labeled in black, with black arrows highlighting prominent peak shifts within the detection time window. Weak spectral features can be better seen in the probe-dependent fits and global analysis plots. For the methanol data, the spectra shown have an average of five pre-excitation spectra subtracted to remove the nonzero background before the actinic pump (see Figure S5).

The dynamics of the protonated molecules after 267 nm excitation in water reveal clear differences in the behavior of nitrophenols, particularly the fact that relaxation for 2NP and 4NP is now quite different (Figure 6a,b), despite the similarity observed when the molecules are deprotonated (Figure 2d,e). The obvious chemical difference is that 2NP is capable of ESIPT, whereas 4NP can undergo ESPT [12,50,51]. Generally speaking, 2NP and 2NP^−^ have qualitatively similar features following 267 nm excitation: a broad ESA, followed by a feature containing a red SE (2NP in Figure 6a, 2NP^−^ in Figure 2d); this pattern lends support to the existence of an intramolecular H-bonding chain between the phenolic hydroxy and nitro groups [14,15], so the photoinduced H-bond breaking in 2NP can lead to TA features resembling 2NP^−^ (i.e., deprotonation at the hydroxy site). The broader ESA and SE bands for 2NP than 2NP^−^ are indicative of ESIPT in action, involving ultrafast intramolecular proton motions in the excited state that intrinsically increase the conformational inhomogeneity of 2NP after photoexcitation [51,52,53]. Not visible on the TA data contour plot due to low signal strength, the global analysis (Figure S6a) and probe-dependent fits (Figure S7a, blue trace) of 2NP in water show a broad positive band (after the ultrafast ESIPT step) that decays with a 1.1 ps lifetime, likely an HGSA band with a similar lifetime to the corresponding nitrophenolate (Figure S2a,d). Moreover, long-lasting red absorption (primarily at wavelengths redder than 550 nm; see the cyan trace in Figure S6a) with a lifetime of ~900 ps is also similar to its counterpart in 2NP^−^ after 267 nm excitation in water (Figure S2d).

In its protonated form, 4NP has the same initial excited-state features as the deprotonated molecule in water: a broad ESA is followed by an overlapped SE band (Figure 2e and Figure 6b). The lifetimes of these features are largely insensitive to the protonation state of the molecule, but the absorption and emission bands are much broader when protonated, indicating that an ESPT pathway may be opened for 4NP in water, and the “vibrationally” hot product after an ESPT reaction from the phenolic hydroxy group could contribute to broader TA bands than those in nitrophenolates [36,54]. After the first two features, the 4NP dynamics significantly change from 4NP^−^ (Figure 2e), with a very long absorption band that narrows and blue-shifts as it decays (Figure 6b). Global analysis fits this decay with two components with lifetimes of ~2.1 and 36 ps (Table 3), which are significantly longer than the 1.4 ps HGSA decay time constant of 4NP^−^ (Table 1). The first feature has significant broad absorption at wavelengths greater than 530 nm, which decays rapidly in the second feature (see Figure 6b and Figure S6b). This pattern leads to the tentative assignment of the first feature to a lingering ESA feature (primarily at >530 nm) along with HGSA (see the consistent blueshift of the peak below 500 nm), and the 36 ps component could involve molecular rotational diffusion in the ground state [32,55]. In addition, there is a long-lived positive absorption band across the entire spectra with a peak below 450 nm showing a lifetime of 4.3 ns, which could involve a nascent triplet state accessible from S_1_′ following ESPT.

Reminiscent of 3NP^−^ in water, 3NP mainly exhibits a prominent yet transient positive band in the excited state (Figure 6c). This feature appears immediately upon excitation and does not blue-shift as it decays, so it is assigned to an ESA that matches the observation for 3NP^−^. Here, ESA decay is fitted via global analysis as a biexponential decay, with ultrafast (<0.1 ps) and 0.8 ps decay components (Figure S6c). The longer decay component of the ESA feature of 3NP (0.8 ps) versus 3NP^−^ (0.6 ps) suggests that CT recombination takes longer when the CT magnitude is reduced (i.e., –OH in 3NP is a weaker electron donor than –O^–^ in 3NP^−^). There is also relatively long-lasting broad absorption with a 140 ps lifetime (Figure S6c) that lacks a clear positive peak below 450 nm, as seen in 4NP (Figure S6b).

**Figure 7:**
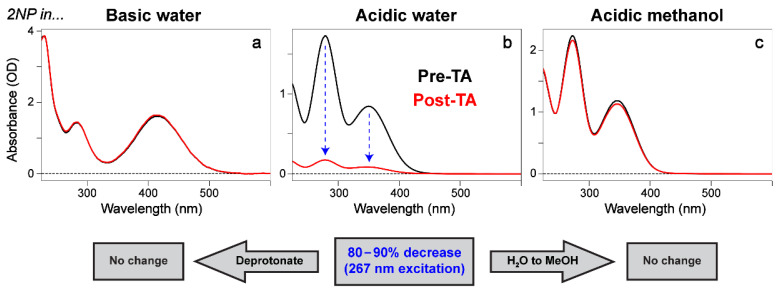
Changes in the electronic absorption spectra of 2NP after fs-TA experiments with 267 nm excitation in (**a**) basic water, (**b**) acidic water, and (**c**) acidic methanol. The baseline from the 550–600 nm region was subtracted from all the spectra for a more accurate comparison. The significant signal reduction magnitude in acidic water is ~80–90%, but is not uniform across all the wavelengths covered. The other two conditions show no clear change in the absorption spectra after UV irradiation on the hour timescale (each TA experimental span).

Perhaps the most significant result was somewhat accidental: the 2NP sample in acidic water degraded due to photoexcitation in TA measurements, which is confirmed by the notable absorption peak intensity decrease (ca. by 80–90%; see Figure 7b) after the TA experiment. Since the TA data were averaged over five consecutive sets, we can compare the sets sequentially to track any changes in dynamics during the experiment (Figure S8). The comparison reveals that the retrieved lifetimes change very slightly and the amplitudes of various features change to some degree (e.g., the SE band is less prominent in set 1, but most prominent in set 5). Relevant comparisons and global analysis performed on the subsets (Figure S8) show further details on the comparisons. Therefore, the average of all datasets was used for most analyses to ensure the best signal-to-noise ratio, particularly since the TA signal strength was low to begin with due to the chromophore solubility, sample absorbance, and laser power limitations due to light conversion efficiencies in the UVC region (267 nm in this work).

This result is of particular interest due to the environmental implications. There have been many previous calculations and experiments on the photolysis of 2NP upon UV excitation, forming HONO in the gas phase due to the high relevance to the atmospheric science, wherein HONO formation is a critical process (see Section 1 above). However, our current work demonstrates that photolysis can be directly observed in solution with a macroscopic spectral signature (not with a minuscule quantum yield), catalyzed only by UV light [56,57,58]. This finding provides strong evidence that ESIPT occurs in 2NP in water (Figure 7b), which is essentially inhibited in methanol (Figure 7c). Even though ESIPT may still occur to some extent in methanol, HONO dissociation may become hindered due to a barrierless reverse ESIPT reaction [53,59,60,61]. Looking forward, due to the nature of photolysis products with low accumulated quantity (possibly a result of quick mineralization of the photolysis products), low transition oscillator strength, or poor resonance conditions that likely preclude their discernible TA signatures, future work should focus on the characterization of nascent species formed via UV-light-induced 2NP transformation/decomposition, and whether it can be accelerated or enhanced in water.

### 2.7. Fs-TA Spectroscopy of Nitrophenols in Methanol after 267 nm Excitation

Surprisingly, TA measurements of the nitrophenols in methanol yield strikingly different spectral patterns versus the largely similar patterns observed for nitrophenolates in both water and methanol, as well as nitrophenols in water. For those aforementioned measurements in general, 2NP and 4NP exhibit similar dynamics, with both having ESA and SE (due to TICT) features, as well as an ensuing well-defined HGSA band. For comparison, 3NP exhibits a longer ESA band, no clear SE features (consistent with the absence of a prominent TICT state; see Section 2.4 and Section 2.5), and a weak HGSA band. In sharp contrast, TA data for the nitrophenols in methanol manifest a very different pattern: 2NP now has unique dynamics (Figure 6d), while 3NP and 4NP are more similar to each other (Figure 6e,f).

In methanol, 2NP can be fitted with an initial strong ESA feature that rapidly decays (240 fs lifetime) into a broad weak absorption with a 5.5 ps lifetime. Both features are attributed to ESA (Table 3) due to the broad absorption and lack of change in their absorption profiles (Figure S6d). The final long-lasting feature (390 ps lifetime) has both a broad red absorption band and a small peak around 450 nm (Figure S6d). Notably, no SE feature is found in any fitting method for 2NP in methanol after 267 nm excitation, while SE is found in all other 2NP-related data in this study (i.e., 2NP in water (Figure 6a and Figure S6a); 2NP^−^ in water and methanol (Figure 2a,d,g and Figure S2a,d,g)). This key result indicates that the ESIPT of 2NP is essentially blocked in methanol; hence, no significant nitro-group twisting motions can occur (i.e., the nitro group is essentially “locked” by the intramolecular H-bonding chain, –OH···ONO) to efficiently return the “hot” chromophore back to the electronic ground state via a TICT state and S_1_/S_0_ CI (see Figure 5a); this is also corroborated by the macroscopic observation of the lack of photodegradation of 2NP in methanol (Figure 7c). Furthermore, if any ESIPT does occur, the potential HONO dissociation is likely inhibited as well, not only due to steric hindrance around the *ortho*-substituents, but also owing to the proposed involvement of triplet states for the HONO release [14,18], which is likely affected by solvent polarity [39]. Future investigations with higher temporal and spectral resolutions, aided by more control samples and higher-level calculations, could shed more light on the interplay between the initial ultrafast ESIPT step, nitro-group rotation, the transient SE band in the TA spectra, and the energy dissipation pathways of 2NP in solution.

4NP and 3NP in methanol show unusual dynamics (Figure 6e,f), especially in comparison to previous data for those molecules (e.g., Figure 6b,c and Figure 2e,f in water, and Figure 2h,i in methanol), likely due to ESPT being blocked in methanol. Both molecules exhibit an initial broad ESA band, though it is faster in 3NP than in 4NP, consistent with more prominent CT characters in 3NP. Next, instead of a negative SE feature followed by a positive feature (ESA or HGSA), they both manifest two broad positive bands. The first band is weaker than the second for both molecules, leading to a second maximum in absorption (~3.4 ps for 3NP, and ~30 ps for 4NP; see green traces in Figure S7e,c). For 3NP, these features blue-shift strongly during the intensity decay (Figure 6f), in accord with slow biexponential HGSA decay, fitted via global analysis with lifetimes of 4.3 and 5.0 ps (Figure S6f). These processes likely involve the solvation dynamics, which are more prominent for the excited-state 4NP in methanol (Figure S6e, blue to green traces) and the hot ground-state 3NP in methanol (Figure S6f, blue to green traces). Once again, we can reason that 4NP exhibits less CT and remains in the excited state for a longer period, thus allowing for solvation of the excited-state population. In contrast, 3NP has stronger CT, leading to faster excited-state decay, and as a result, the solvation occurs in the HGS. Subsequently for 3NP, there is a long-lived feature with both a peak below 450 nm (likely HGSA) and a broad red absorption (likely a trapped state), associated with the retrieved lifetime of 810 ps (Figure S6f).

This model is more complicated for 4NP, since the two positive features after ESA have much longer lifetimes (12 and 37 ps) than 3NP (4.3 and 5.0 ps). In addition, there is no consistent blueshift for 4NP, but instead, a slight redshift in the middle of the dynamics (from ~500 fs to 10 ps; see Figure 6e) due to the specific excited-state energy gap change pattern (e.g., a steeper slope of the S_n_ PES could occur to lower the upward transition energy gap). This finding suggests that the pertinent relaxation is still dominated by ESA, since HGSA would blue-shift as the hot S_0_ state relaxes toward the thermally equilibrated structure. Next, the decay process after 10 ps exhibits a strong blueshift (typically HGS relaxation) for 4NP. From global analysis, the absorption profile of the final feature (Figure S6e) more resembles an HGSA feature than the weak, broad, red absorption observed for the long-lasting absorption in other samples’ spectral signatures. We can thus tentatively assign these final features to HGSA. As a result, the molecule becomes solvated in the excited state for ~10 ps, and then, crosses over (via internal conversion other than an S_1_/S_0_ CI rapidly accessed via a prominent TICT state; see above) and relaxes in the ground state on the hundreds of ps-to-ns timescale, which may involve some of the small-scale nitro-group back/reverse twists to the original ground state [23,62]. The HGSA then blue-shifts until the end of the measurement window, 900 ps after the initial excitation. The final feature retrieved from global analysis is blue-shifted absorption with a peak at ~470 nm and a 1.3 ns lifetime (Figure S6e); however, this feature seems to mainly capture the blueshift of the HGSA (likely encountering a small ground-state barrier, hence the lengthened lifetime; see Table 3 for comparisons to 2NP and 3NP in methanol) rather than a trapped excited state with a much weaker positive band, as observed in most other nitrophenols and nitrophenolates under 267 nm excitation (Figure 2 and Figure 6). Notably, the three-pulse FSRS technique has been implemented to track ultrafast vibrational cooling processes from the excited state to the ground state [36,63,64,65], which can be implemented for future studies of the unusual spectral features of 4NP in water and methanol with significantly longer time constants (see Figure 6b,e and Figure S6b,e) than those of 2/3NP (Figure 6a,c,d,f and Figure S6a,c,d,f). These targeted vibrational measurements may reveal further mechanistic insights into the electronic ground-state PES with the proposed intermediate energy barrier(s) and much-lengthened lifetimes.

### 2.8. FSRS Signatures of Nitrophenols and Nitrophenolates in Water

All the spectroscopic characterization so far concerns the electronic structure of nitrophenols and nitrophenolates, yet the nuclear coordinates constitute an indispensable part of the multidimensional PES to fully describe the structure–function relationship [66,67,68]. Raman spectroscopy provides a unique spectral toolset to structurally characterize the site-specific vibrations of small molecules in aqueous solution. In particular, no solvent deuteration (i.e., D_2_O) is needed, as the HOH bending mode, despite being dominant around 1650 cm^–1^ in IR spectroscopy, displays minimal Raman intensity [69]. A recent development in Raman methodology, termed femtosecond stimulated Raman spectroscopy (FSRS) [54,70,71,72,73,74,75,76,77,78], is a powerful technique that can resonantly enhance the typically weak Raman scattering signal while allowing for certain species to be isolated by strategically tuning the Raman pump wavelength to desired regions [54,75], either in the electronic ground [79] or excited states [43,80] depending on the laser pulse combination and sequence used. There are a few dispersed reports of theoretical and experimental Raman spectra of nitrophenols in solution [81,82]; however, a unified high-quality investigation comparing the vibrational spectral signatures (“fingerprints”) of nitrophenols and nitrophenolates in water has not been reported to the best of our knowledge.

**Figure 8:**
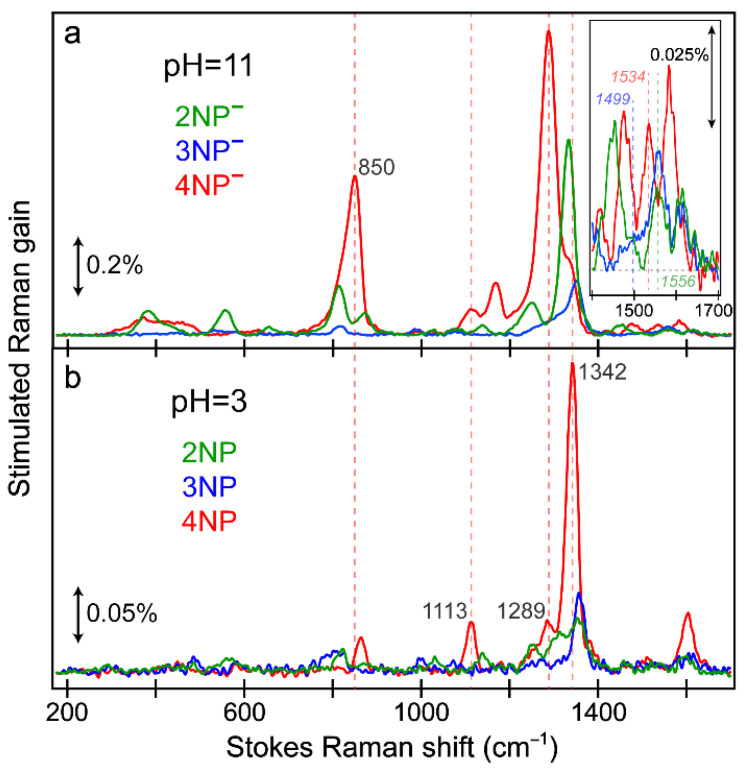
Ground-state FSRS spectra of various nitrophenolates and nitrophenols in water. (**a**) FSRS spectra of 2NP^−^ (green), 3NP^−^ (blue), and 4NP^−^ (red) are overlaid. The stimulated Raman gain magnitude of 0.2% is denoted by the double-arrowed line. The high-frequency region above 1400 cm^–1^ is enlarged and shown in the inset on the right side, with color-coded vertical dashed lines denoting the C=O stretching modes in three nitrophenolates, as aided by quantum calculations (see Figures S9 and S11). (**b**) FSRS spectra of 2NP (green), 3NP (blue), and 4NP (red) are overlaid. The stimulated Raman gain magnitude of 0.05% is denoted by a double-arrowed line. Vertical red dashed lines highlight the peak frequency variation between the samples, with key frequencies labeled.

The ground-state (GS)-FSRS spectra on the Stokes side of the nitrophenols in acidic water (pH = 3) and nitrophenolates in basic water (pH = 11) were collected (Figure 8) with a Raman pump wavelength of 540 nm. We remark that the pre-resonantly enhanced GS-FSRS measurements allowed us to obtain the aforementioned vibrational fingerprints in the electronic ground state, while a direct measurement of excited-state processes requires the incorporation of a preceding actinic pump [54,70,75] or a Raman pump that overlaps with the ground-state absorption band [79,80,83]. Herein, the red-shifted ground-state absorption of the deprotonated chromophores (Figure 1) leads to better pre-resonance enhancement of the Raman peaks relative to their protonated counterparts. Among the three nitroaromatic molecules at a concentration of 1 mg/mL, 4NP has the largest ground-state absorption intensity, leading to the largest Raman peaks under high and low pH conditions. The nitrophenolate GS-FSRS spectra are most similar for 2NP^−^ and 4NP^−^ (e.g., highlighted by the high-frequency region in the Figure 8a inset), with both molecules displaying a largely conserved peak pattern, as expected given the similarity between the electronic effects of an electron-withdrawing substituent (–NO_2_) at the *ortho*/*para* positions [50]. This observation is supplemented by the similar p*K*_a_ values of 2NP and 4NP (Figure S1d,e) compared to 3NP (Figure S1f). While the pH values of the acidic (basic) solutions are significantly lower (higher) than the measured p*K*_a_ values (Figure S1), evidence for the remaining protonated 4NP chromophores in the pH 11 solution (Figure 8a) is highlighted using dashed lines in Figure 8. Similarly, evidence for some of the remaining deprotonated 4NP population can be seen in the pH 3 solution (Figure 8b).

The comparisons between the experimental and calculated Raman spectra of three nitrophenolates (Figure S9) and three nitrophenols (Figure S10) in water are accompanied by their corresponding vibrational normal mode assignments (Tables S3–S5 for nitrophenolates and Tables S6–S8 for nitrophenols). Interestingly, many of the calculated Raman frequencies do not need to be scaled to match the experimental frequencies, especially those including intense vibrational peaks between ~1250 and 1350 cm^–1^. Notably, evidence for varying degrees of charge-transfer (CT) character emerges upon inspecting the peak frequencies and mode assignments of the nitrophenolates. The experimental peak frequencies assigned to a dominant C=O stretch for the nitrophenolates follow a consistent trend: 1556 > 1534 > 1499 cm^–1^ for 2NP^−^ > 4NP^−^ > 3NP^−^ (Tables S3–S5). We note that the experimental peak for 3NP^−^ is rather weak as a shoulder versus the other nitrophenolates (see Figure 8a inset), but the pertinent vibrational normal mode is consistent with DFT calculation results as Raman activity for the 3NP^−^ C=O stretch is low (Figure S9b), which can also be inferred from the significantly reduced nuclear displacement of the C=O bond (Figure S11, top middle panel) versus the other nitrophenolates (Figure S11, top left and right panels). Meanwhile, the peak frequencies assigned to a dominant adjacent (see Figure 1 insets for chemical structures) C–N stretch follow the opposite trend: 1350 > 1287 > 1249 cm^–1^ for 3NP^−^ > 4NP^−^ > 2NP^−^. As –C=O^(−)^ is considered to be a strong electron donor, while –NO_2_ is amongst the strongest electron-withdrawing groups due to resonance, the aforementioned peak frequency patterns paint a vivid picture of the CT strengths “inside” the nitrophenolates. 2NP^−^, with the bluest C=O stretch and reddest C–N stretch, possesses the weakest CT character in contrast to 3NP^−^, with the most prominent CT character according to the fact that it has reddest C=O stretch and bluest C–N stretch frequencies. The strong CT character of 3NP^−^ is in accord with a prior investigation on the electronic effects of the substituent position on nitrophenolate isomers [12] and our systematic fs-TA spectral analysis, which does not result in an HGSA for 3NP^−^ only (Figure 2 and Figure 3). This point may be the reason for the relatively poor match between the calculated and experimental Raman spectra for 3NP^−^ (Figure S9b), since density functional theory (DFT) calculations are known for being ineffective in predicting and encapsulating CT states [84,85]. This finding is also supported by the consistent mismatch between experimental and theoretical vibrational peak frequencies between ~1250 and 1350 cm^–1^ (primarily involving C–N stretch) for all three nitroaromatic molecules (Figure S9).

The aforementioned mismatch between the calculated and experimental Raman peaks is significantly reduced for the nitrophenol spectra (Figure S10), likely due to the reduced CT character with the neutral hydroxy substituent (–OH) opposed to the anionic nitrophenolate chromophore. Notably, the calculated Raman frequency primarily assigned to C–N stretch remains redder than the experimental peak, hinting that DFT calculations still struggle to capture the “weaker” CT state (with less CT magnitude than that in nitrophenolates). Upon inspection of the nitrophenol modes assigned to C–N stretch, a similar pattern to that of the nitrophenolates is observed: 1359 > 1341 > 1309 cm^–1^ for 3NP > 4NP > 2NP (Tables S6–S8). The reduced signal intensity and lack of a clear –COH stretch for the nitrophenols do not allow for analysis of the other substituent; however, the trend of the “acceptor” C–N stretch frequency confirms that 3NP has the strongest CT, while 2NP has the weakest CT, regardless of the protonation state. Interestingly, upon comparing the C–N stretching frequency for the protonated versus deprotonated chromophores, the protonated frequency slightly blue-shifts from 3NP^−^ to 3NP (1350→1359 cm^–1^), while a significantly larger blueshift is observed for 2NP (1249→1309 cm^–1^) and 4NP (1287→1341 cm^–1^). The blue-shifted C–N stretching frequency of the protonated chromophores, given the reduced electron-donating strength of C–OH versus –C=O^−^, hints at the complexity/intricacy of the equilibrated CT magnitude in the ground state versus the light-induced CT character in the excited state (see above). However, the disparity between the shift magnitude for 3NP (9 cm^–1^) versus 2NP (60 cm^–1^) and 4NP (54 cm^–1^) suggests that the “*meta*” effect relies more on the substituent position (of the specific regioisomer), while the donating strength of the substituent plays a smaller role. In contrast, the donor strength of the substituent at the *ortho*/*para* positions plays a much larger role in 2NP and 4NP.

The detailed analysis of these Raman peaks can offer two additional avenues of continued research, aside from the fundamental investigation of the influence of structural modifications on the electronic properties. First, what roles do these vibrational motions play in the propensity for ESIPT in 2NP? Second, advanced Raman spectroscopy can be further developed and implemented as an effective and efficient sensing technique to determine nitrophenol(ate) concentrations in water systems, rainwater, and the atmosphere. The role ESIPT may play in the photolysis of 2NP is an ongoing area of research, and it has been speculated that ESIPT inside 2NP can lead to nitrous acid (HONO) formation and dissociation that could be coupled to certain vibrations. Notably, the ~881 and 288 cm^–1^ Raman modes of 2NP in water involve motions of the –NO_2_ and C–OH moieties (see Table S6) that bring them into close proximity to facilitate proton transfer [36,51,86,87]. These motions, along with several others (1613, 1552, 1466, 833 cm^–1^), will be the focus of future excited-state (ES)-FSRS measurements of 2NP in acidic water. We envision that ultrafast Raman spectroscopy, with its unique and field-proven advantages over conventional Raman spectroscopy [36,54,67,70,71], can be developed into a sensitive technique to not only quickly and easily detect nitrophenols in the environment but also to differentiate between variously substituted nitrophenols and nitrophenolates in waterways by characterizing their distinct vibrational “fingerprints”.

## 3. Discussion

The combination of all these time-resolved electronic spectroscopic results demonstrates that the protonation state and solvent play an important role in the dynamics of the series of NP and NP^−^ samples. When the molecules are protonated (i.e., 2/3/4NP), potential ESIPT and ESPT processes complicate the electronic and structural dynamics in both solvents (water and methanol). When the molecules are deprotonated (i.e., 2/3/4NP^−^), the dynamics for each molecule differ only slightly between methanol and water. The HGSA lifetimes are increased for all molecules in methanol, mainly due to weaker solvent–solute interactions, longer solvation time, and less effective accommodation of the CT/TICT states versus water. The excited-state component lifetimes also increase slightly in methanol. In particular, we identify clear groupings in the electronic dynamics based on the substituent placement. The *ortho-* and *para*-substituents, with access to similar electronic resonance structures, exhibit similar dynamics and are different from the *meta*-substituent dynamics. This finding suggests that charge transfer plays a dominant role in excited-state energy relaxation, and the difference caused by the relative substituent placement is the most significant factor, which is further corroborated by the p*K*_a_ (ground-state acidity) and GS-FSRS (vibrational structure) results. An interesting finding is the brief delay of the prominent ESA band observed in water but not in methanol, which is attributed to the early-time excited-state dynamics with a miniscule yet discernible ESA band (Figure 3b,d,f and Figure 5).

In water, the protonation state can exert varying effects. For 2NP, the observed dynamics are similar to 2NP^−^, except for the fact that 2NP breaks down under UV light irradiation, likely involving ESIPT and the HONO formation (see above) [9,56]. For 4NP, the excited-state features are similar in both protonation states, but the HGS relaxation is significantly lengthened, and a new, long-lasting absorption band across the entire spectral window is found, in accord with ESPT and the formation of a “hot” photoproduct for 4NP (but not for 4NP^−^). For 3NP, the protonated molecule has almost the same features as the deprotonated molecule (so ESPT could also occur), but with shorter lifetimes. This result can be attributed to the *meta*-effect, as the elevated p*K*_a_ of 3NP (versus 2/4NP; see Figure S1) leads to its reduced (photo)acidity and less pronounced ESPT; meanwhile, the charge recombination timescale of the ICT state (e.g., see 3NP^−^ in Figure 5b) could be lengthened in the photoexcited 3NP due to the interplay between the ICT magnitude (less in 3NP) and H-bonding strengths (less in 3NP) of both the –OH and –NO_2_ groups to the adjacent solvent molecules, which is even further lengthened in methanol versus water.

Furthermore, the solvent has a much more significant effect on dynamics when the molecule is protonated due to the participation of ESPT/ESIPT, which is especially prone to being affected by specific solvent molecules [32,36]. Comparing the nitrophenols in water and methanol, the dynamics observed in methanol are substantially different for each molecule. In addition, the “grouping” similarities between molecules change. Instead of the *ortho*- and *para*-isomers being similar, with differences upon comparison to the *meta*-isomer, in methanol, the *ortho*-isomer becomes different while the *meta*- and *para*-isomers are more similar. These results suggest that instead of the electronic effects being the dominant factor, the nuclear distance between the nitro and hydroxy groups can play a more important role when the molecules are protonated and in different solvents, wherein solvent molecules can affect the intramolecular H-bonding chain either directly (e.g., forming intermolecular H-bonds) or indirectly (e.g., via electrostatic or steric interactions). In particular, the disruption of intramolecular H-bonding plays a more prominent role for 2NP due to the proximity between the hydroxy and nitro substituents, while a longer-range H-bonding chain could occur for 3/4NP (see the chemical structures in Figure 1).

There is also an interesting solvent relationship with the observed photolysis of 2NP. It is considered that the photolysis occurs due to ESIPT from the hydroxy group to the nitro group, forming an *aci*-tautomer (the nitronic acid form). Therein, the HONO can detach from the molecule in the excited state. In our experiments, UV-laser-induced photolysis was observed in water but not in methanol (Figure 7b,c), suggesting that methanol may hinder either the ESIPT pathway or the subsequent HONO detachment in the solvent. Previous calculations showed that hydrogen transfer from the *aci*-tautomer back to *o*-nitrophenol (2NP) is effectively barrierless in the electronic ground state [14], so conversion to the *aci*-tautomer and relaxation to the ground state would allow for efficient conversion back to the original molecule, especially in methanol because the lengthened HGS relaxation may allow this step to occur. The definitive determination of whether ESIPT occurs in either solvent could inspire future experimental and/or computational work, possibly on excited-state FSRS characterization [51,86,88], which underlies the importance of fundamental mechanisms and potential applications for these environmentally relevant molecules to achieve desirable outcomes and societal benefits. In essence, we envision the systematic characterization of nitrophenols and nitrophenolates in water and alcohol (methanol) in this comprehensive work to lay a solid foundation for future steady-state and time-resolved (particularly ultrafast) spectroscopies of these environmentally relevant small molecules in condensed phase, including both excited-state FSRS [54,75,78] and its time-domain analogue (impulsive Raman or ISRS (impulsive stimulated Raman scattering) [89,90,91]). The high temporal resolutions for nonlinear Raman techniques, either in the mixed time-frequency domain, such as FSRS, or purely in the time domain, such as ISRS, are desirable and necessary (versus spontaneous Raman schemes) to investigate excited-state processes with vibrational specificity (i.e., simultaneously high spectral resolution). Such a line of inquiry will continue to inspire a broad community of scientists and engineers to utilize the elucidated excited-state molecular mechanisms to efficiently engineer or treat them for a clean and sustainable world.

## 4. Materials and Methods

### 4.1. Sample Preparation

All three nitrophenols were purchased from TCI America, Inc. (Portland, OR, USA). 2-nitrophenol (2NP, *ortho*-isomer) and 3-nitrophenol (3NP, *meta*-isomer) have >98% purity (GC—gas chromatography), while 4-nitrophenol (4NP, *para*-isomer) has 99% purity; these were all used without further purification. The HPLC-grade dry methanol was purchased from Fisher Scientific, and the deionized (DI) Millipore water was used for the experiments performed in water. For the spectroscopic measurements, buffer solutions were used to maintain a stable pH. The low-pH buffer (pH = 4) used an acetic acid and sodium acetate buffer in water, while the high-pH buffer (pH = 10) used a sodium bicarbonate and potassium hydroxide buffer. Slightly different pH values (~3 and 11) were used for GS-FSRS measurements to be certain of the protonated and deprotonated chromophores (see Figure 7). For the measurements in acidic and basic methanol solution, acetic acid and 1,8-diazabicyclo [5.4.0]undec-7-ene (DBU) were added (0.5% by volume), respectively. Similarly, for the extra control experiment for 3NP^−^ in acetonitrile (see Figure S1 appendix figure), ~0.5% (*v*/*v*) DBU was used. For the p*K*_a_ measurements, a variety of buffers were used to cover a broad pH range. Buffers of acetic acid/sodium acetate, monopotassium phosphate/potassium hydroxide, hydrochloric acid/tris base, and sodium bicarbonate/potassium hydroxide were used to achieve the solution pHs in the ranges of 4 to 5.5, 6 to 7, 7.5 to 9, and 9.5 to 10, respectively.

### 4.2. Steady-State Electronic Absorption Measurements

A Thermo Scientific Evolution 201 UV/Visible (UV/Vis) spectrophotometer was used to measure the ground-state electronic absorption spectra of 2NP, 3NP, and 4NP in acidic (pH = 4) buffer solution and 2NP^−^, 3NP^−^, and 4NP^−^ in basic (pH = 10) buffer solution, housed in a 1 mm pathlength quartz cuvette (Spectrosil 1-Q-1, Starna Cells, Inc., Atascadero, CA, USA) at room temperature (~72 °F). The spectrophotometer was similarly used to determine the p*K*_a_ values of the nitrophenols in solution. A consistent amount of sample was dissolved in a series of buffer solutions (i.e., identical sample concentration), the pH values were measured, and the electronic absorption spectra were measured for each condition. From these spectra, the pH-dependent amplitude of the most prominent peak in association with each protonation state was used to calculate the molar fraction of the sample in each protonation state. Experimentally, as a first step, each of the nitrophenol samples was dissolved in a stock solution of deionized water, and an identical amount of the solution was mixed into a buffer solution prepared for a specific pH. The pH value and UV/Vis spectrum of the resulting solution (including the nitrophenol) were then measured in a systematic manner [92]. From the spectra, characteristic wavelengths were selected for the protonated and deprotonated species based on major peaks (see Figure S1a,b,c in Supplementary Materials). For example, for 2NP, the peak wavelengths of the protonated species (*λ_A_*) and deprotonated species (*λ_B_*) are 351 and 416 nm, respectively. The measured absorption intensity changes at these wavelengths enabled the quantitative calculation of the molar fraction of each species according to the experimental formulae:

(1) $$R_{A}=\frac{A\left(\lambda_{A}\right)-A^{min}\left(\lambda_{A}\right)}{A^{max}\left(\lambda_{A}\right)-A^{min}\left(\lambda_{A}\right)}$$

(2) $$R_{B}=\frac{A\left(\lambda_{B}\right)-A^{min}\left(\lambda_{B}\right)}{A^{max}\left(\lambda_{B}\right)-A^{min}\left(\lambda_{B}\right)}$$

where the subscript *A* indicates the protonated form, *B* indicates the deprotonated form, the function *A(λ)* represents the absorbance at wavelength *λ*, and the *max* and *min* superscripts denote the maximal and minimal absorbances at the wavelength measured across all the pH conditions. To obtain the p*K*_a_ of each sample, the two-form molar fractions at each pH (see red and black data points in Figure S1d,e,f) can be fitted using a least-squares fitting procedure according to the two ensuing functions of the theoretical molar fractions at each pH:

(3) $$R_{A}=\frac{1}{1+10^{\left(pH-pK_{a}\right)}}$$

(4) $$R_{B}=1-\frac{1}{1+10^{\left(pH-pK_{a}\right)}}$$

where $R_{A}$ is the molar fraction that is protonated, $R_{B}$ is the molar fraction that is deprotonated, and the p*K*_a_ value can be derived from the fitting process. The reported p*K*_a_ is the average of the two best fits covering the decreasing protonated population and increasing deprotonated population simultaneously. The p*K*_a_ for each species, as well as the crossing point ($R_{A}=R_{B}=0.5$; hence, pH = p*K*_a_) of the two fitted lines, was used to determine the p*K*_a_ with an accuracy of 0.1 or less. Note that this procedure was not performed to achieve an ultrahigh-precision p*K*_a_ measurement; it was performed to verify the previously measured p*K*_a_ values [2], and to make sure that the pH conditions used for our steady-state electronic absorption, GS-FSRS, and fs-TA experiments were sufficiently high or low to measure only the deprotonated or protonated species, and to avoid complications due to excessive chromophore inhomogeneity.

### 4.3. Femtosecond Stimulated Raman Spectroscopy (FSRS) and Femtosecond Transient Absorption (fs-TA) Spectroscopy

The home-built tabletop FSRS and fs-TA optical setups begin with a mode-locked Ti:Sapphire oscillator (Mantis-5) and a regenerative amplifier (Legend Elite USP 1K HE, Coherent, Inc., Santa Clara, CA, USA) that provides a ~35 femtosecond (fs) pulse centered at ~800 nm with an average power of 3.6 W and a repetition rate of 1 kHz. The generation of a tunable picosecond (ps) Raman pump consists of four major components: an fs noncollinear optical parametric amplifier (NOPA) to generate a tunable broadband seed, a grating-slit-based spectral filter to produce a ps narrowband seed, a second harmonic bandwidth compressor (SHBC) to generate a ps 400 nm pump, and a two-stage ps-NOPA system to amplify the ps seed and achieve a tunable Raman pump with sufficient power. This sophisticated setup has been described in detail in previous publications [46,93,94]; however, a brief description regarding the generation of the ps 540 nm Raman pump for GS-FSRS measurements on the Stokes side for nitrophenols in solution is provided below.

The fs-NOPA involves the combination of a white-light seed and 400 nm pump beam on a beta-barium-borate (BBO) crystal to generate a tunable fs laser beam from ~480 to 720 nm. The spectral filter selects a narrow portion of the aforementioned tunable fs pulse, effectively producing a lengthened ps pulse at the desired wavelength. This process is quite inefficient; hence, a two-stage ps NOPA is required to generate a Raman pump of appreciable power for GS-FSRS with sufficient signal-to-noise ratios. The SHBC introduces positive and negative chirps to two portions of the fs 800 nm fundamental beam to temporally stretch the pulses before they are overlapped onto a BBO crystal to generate ps 400 nm pulses. The spectral filter output and ps 400 nm pump are spatially and temporally overlapped onto the BBO crystals in the subsequent two-stage ps-NOPA system to generate the ps Raman pump. The Raman probe is supercontinuum white light (SCWL) generated by focusing a portion of the fundamental beam onto a water-filled 2 mm thick quartz cuvette (Spectrosil 1-Q-2, Starna Cells, Inc., Atascadero, CA, USA). The Raman pump and probe are spatially and temporally overlapped onto the sample in a 1 mm thick quartz cuvette (Spectrosil 1-Q-1, Starna Cells, Inc., Atascadero, CA, USA) with a pump laser spot size of ~0.2 mm in diameter at the focal point [95,96]. The transmitted probe is then directed into an imaging spectrograph (IsoPlane SCT-320, Princeton Instruments, Inc., part of Teledyne Princeton Instruments, Trenton, NJ, USA) with a CCD array camera mounted at the exit focal plane (PIXIS:100F, Princeton Instruments, Inc., Trenton, NJ, USA). For GS-FSRS measurements on the nitrophenols, the Raman pump power was set to ~5 mW, while the nitrophenol concentration in the low- and high-pH buffer solutions was set at 1.0 mg/mL. The sample solutions were constantly stirred using a magnetic stir bar during time-resolved laser spectroscopic measurements to ensure sample stability under femtosecond laser irradiation.

To generate the 267 nm actinic pump pulse for fs-TA measurements, third harmonic generation (THG) was achieved using a FemtoKit (EKSMA Optics, UAB) consisting of two 1 mm thick BBO crystals, one zero-order dual waveplate at 800 nm (*λ*/2) + 400 nm (*λ*), and one 1.7 mm thick calcite group-velocity-delay compensation plate [37,97]; then, the transmitted light was selected using a dichroic mirror with high reflectance and transmittance at 257–275 and 400 + 800 nm, respectively. The 267 nm actinic pump pulse was not further temporally compressed following THG generation, resulting in a pulse duration of ~200–300 fs. A portion of the fundamental was focused onto a BBO to generate the 400 nm actinic pump pulse via second harmonic generation (SHG), subsequently compressed via a UV-grade fused silica ultrafast Brewster-angle dispersing prism pair (06SB10, Newport, Inc.) to a pulse duration below 100 fs [94,98]. The white light probe for fs-TA measurements was generated in a similar manner to the Raman probe using temporal compression via a chirped mirror pair (DCM-12, 400–700 nm, Laser Quantum, Inc., part of Novanta, Stockport, UK). Similar to GS-FSRS measurements, the actinic pump and probe pulses were overlapped onto a 1 mm thick sample cuvette before directing the transmitted probe pulse to the spectrograph and CCD camera. For the 267 nm fs-TA measurements, the actinic pump power was set to ~0.3 mW. The nitrophenol samples were flowed through a quartz cell (48-Q-1, Starna Cells, Inc., Atascadero, CA, USA) using a home-built miniature peristaltic pump to ensure sample stability under the pulsed laser irradiation, while the optical density (OD) of the nitrophenol samples ranged from 1 to 3 at 267 nm (UVC excitation wavelength). For the 400 nm fs-TA measurements, the actinic pump power was set to 0.6 mW and the OD of the nitrophenol solutions was 0.5–0.7 per mm at 400 nm, while the sample solution was constantly stirred via a magnetic stir bar to ensure fresh sample was irradiated during the TA experiments. All the laser pulses were parallel-polarized to improve the signal-to-noise ratio.

For all GS-FSRS and fs-TA measurements at room temperature, the UV/Vis spectra were measured before and after the experiments to monitor sample stability. To achieve the desired balance between the spectral coverage and resolution of the collected data, the reflective grating inside the spectrograph was set to 1200 grooves/mm with a 300 nm blaze wavelength and 300 grooves/mm with a 300 nm blaze wavelength for the GS-FSRS and fs-TA measurements, respectively.

### 4.4. Quantum Calculations

Calculations on the electronic ground and excited states of the nitrophenolates were performed using Gaussian 16 software (Gaussian, Inc., Wallingford, CT, USA) [44]. Ground-state structures and energies were optimized using the DFT (density functional theory) method with the RB3LYP functional and 6-31G+(d,p) basis sets. The lower level of theory was used to allow extensive scans to be made of both nuclear coordinates (see Section 2.4) since higher levels of theory took significantly more time. Excited-state structures were calculated using the TD-DFT method with the same settings as above. The solvent (water, herein) was approximated using the default IEFPCM (implicit) solvation model. To dissect the excited-state relaxation pathways, a coordinate scan was performed by adjusting the dihedral angle formed between the nitro group and the ring (C–C–N–O), as well as the pyramidalization angle of the nitro group (C–O–O–N) with 15° and 5° step sizes, respectively. The scan was started with the dihedral angle at 90° (i.e., the nitro group plane is perpendicular to the ring plane) and the pyramidalization angle at 0° (i.e., the nitro group and connected carbon are all in the same plane). Note that the depicted ground-state PES in Figure 4 can be labeled as S_0_′ due to its energy being obtained at the nuclear coordinates corresponding to the optimized excited state of S_1_ for various nitrophenolates in water [45].

To calculate the ground-state Raman spectra of the nitrophenols and nitrophenolates in water, the structures of all molecules were first optimized in the ground state (S_0_) using the DFT optimization method in Gaussian 16, with RB3LYP functional [99,100], 6-311G+(d,p) basis sets, and IEFPCM water solvent (an implicit solvent model) [101,102]. Next, the same level of theory and basis sets were used to run ground-state frequency calculation to obtain the off-resonance Raman spectra.

### 4.5. Probe-Dependent Spectral Analysis

Traditional probe-dependent analysis of nitrophenols is complicated by the significant overlap of all spectral features and a clear rise in signals well beyond time zero. For example, a negative SE band emerges after an initial positive ESA feature in 4NP^−^ after 267 nm excitation (see Figure 2e and Figure 3d). Fitting with a parallel model (assuming all decay occurs starting from the same initial time) is thus unfeasible to capture this spectral feature. Analysis of the TA dynamics at specific wavelengths was instead performed by fitting the spectra to a kinetic model of consecutive first-order reactions:

(5) $$exc.\;\left(t_{0}\right)\to x_{1}\to x_{2}\to \ldots \to x_{n-1}\to x_{n}$$

which can be mathematically modeled as a system of ordinary differential equations:

(6) $$\frac{dx_{1}}{dt}=-k_{1}x_{1}$$

(7) $$\frac{dx_{2}}{dt}=k_{1}x_{1}-k_{2}x_{2}$$

(8) $$\frac{dx_{n}}{dt}=k_{n-1}x_{n-1}-k_{n}x_{n}$$

where $x_{1}$ is the population of the first state, $x_{2}$ of the second state, up to $x_{n}$ of the nth state, and the decay constants are given by $k_{1}$, $k_{2}$, up to $k_{n}$, respectively. The initial population ($x_{1}$) was modeled by convolving exponential decay using a gaussian peak to model the actinic excitation process, while the consecutive populations were calculated by numerically integrating the system using Equations (6)–(8).

To fit the observed spectra, each population $x_{i}$ had two parameters: the amplitude ($A_{i}$) and decay rate constant ($k_{i}$). The initial population ($x_{1}$) was convolved using a gaussian peak, and so, it needed two additional parameters: the width of the gaussian peak (the approximate cross-correlation time of the pump and probe pulses) and time zero (the time when the pump and probe pulses are coincident). Any significant baseline/offset was fitted by adding a constant to the model. Note that the rise in each feature is precisely due to the decay of the previous feature, thus eliminating the need to fit an additional rise component parameter for delayed spectral features. This approach gives a total of 3 + 2n parameters for an n-population system.

In particular, the model was fit to TA spectral plots (e.g., Figure 3) using a Python script with a least-squares fitting algorithm after specifying the appropriate initial parameters and ranges of parameters. This procedure did not pre-determine the number of states to use, so it was determined by trial-and-error and via comparison with the results from other analyses (e.g., independent processing, global analysis as described in Section 4.6 below, and a parallel model). For many systems, a purely sequential/parallel model is unlikely [103,104]. A mixture of both is more reasonable; we tried both a parallel model and more complicated kinetic models, but did not obtain more meaningful insights; hence, we used a typical sequential model as a reasonable simplification. Generally speaking, the fewest states possible were used to fit all the key features in the TA data (usually no more than four).

### 4.6. Global Analysis

The global analysis of TA data was performed using the open-source software Glotaran [105]. In general, the fewest parameters necessary to fit all the relevant spectral features was used [103,106]. The excitation was modeled using a Gaussian-profile instrument response function with time zero and the pulse full-width at half-maximum (FWHM) fitted. A linear dispersion function was also used to model the small amount of dispersion (i.e., chirp) in the pump pulse (e.g., ~60 fs with 400 nm pump), which can then be accounted for to clean up the dynamic fits. The time zero of the TA data was also fitted within the global analysis using the “IRF” function in Glotaran, and this parameter was allowed to vary freely during the fitting procedure. Generally, this parameter reports a time value near the experimentally set time zero (within ~100 fs); however, for the measurements of various nitrophenolates in water upon 267 nm excitation, the time zero was fitted with a notably late time of around 400–500 fs. This result may be attributed to Glotaran being unable to fit the very weak early absorption feature, so the fitting only started when stronger features appear in the spectra. Time-zero values were also obtained using the probe-dependent fitting procedure (e.g., Table 2, and Tables S1 and S2) and these values do not always correspond exactly with the global analysis values; this finding occurs because global analysis can capture the entire spectra, as well as any dispersion of the pump pulse that may occur. Between the two methods, the retrieved time-zero values follow the same trends, but for the aforementioned reasons, we consider that the time-zero values from the global analysis are more accurate.

All the TA spectra were fitted assuming a sequential kinetic model (see Section 4.5 above for details, while the more complex target analysis yields similar results). If fits did not converge, the rate constant for the first feature was fixed at 10^14^ s^–1^, corresponding to a lifetime of 10 fs. If they still did not converge, a second rate constant was then fixed at 5 × 10^13^ s^–1^ (20 fs lifetime). These values were chosen within the cross-correlation time to capture the coherent artefacts that are largely irrelevant to the chromophore responses of interest. Other values for the fixed-rate constants were tried, showing little effect on the rest of the fitting. This procedure can be rationalized by the fact that the cross-correlation time of the pump–probe pulses was ~250 fs with uncompressed UV light, so any time constant substantially shorter than that cannot be determined with high accuracy. In the lifetime tables, these short lifetimes are indicated by <0.3 ps to reflect the early-time uncertainty in our measurements; exact values from the global analysis fits are shown in the Supplementary Materials (Figures S2 and S6).

Due to the strong pump–pulse interaction with samples in the 400 nm excitation experiments (see Figure 2 and Figure 3), ultrafast global analysis features were used to account for large coherent artefacts and prevent them from affecting the rest of the global analysis. For 2NP^−^ and 4NP^−^, only one feature was needed. For 3NP^−^, two features were needed. The lifetimes of these features were fixed at 10 fs, except for the second feature for 3NP^−^, which was fixed at 20 fs. The inclusion of these features did not affect the analysis much, but it allowed a clearer depiction of the early features [33]. Notably, the results of our analysis closely match previous results on the same molecular system which accounted for the pump–pulse interaction in a different way [17]. Moreover, there is a clear sharp negative dip at 470 nm around time zero that disappears within ~150 fs, which is seen in all nitrophenolates and other samples (see Figure 2a–c with 400 nm excitation, likely corresponding to a water scattering peak since the energy gap between 400 and 470 nm roughly matches the water O–H stretching band frequency), and thus, is not relevant to the chromophore molecular dynamics.

To plot the retrieved spectral components from the global analysis, the spectral profiles from the species-associated spectra (SAS) plot were multiplied by the amplitude of the estimated concentration as a function of time for each feature reported in Glotaran. Note that the concentration is inherently inaccurate since the pertinent transition oscillator strength of the species is unknown, but it allows for visualization of which species are present in the spectra at each time point. This SAS scaling was performed because the fit generated and the concentration are inherently correlated; hence, the spectral signal amplitude should be a product of the two amplitudes. Usually, this step is not necessary because the concentration amplitudes are all similar and near unity; however, for some of the short-lived features, the amplitude of the concentration can be incredibly small and the SAS feature is thereby displayed as much larger than the observed data. Therefore, to better visualize and compare the spectral features in one plot for each sample (e.g., Figures S2 and S6), we applied this scaling method systematically to present the global analysis results for the series of nitrophenolates and nitrophenols in solution after 400 and/or 267 nm excitation.

## Figures and Tables

**Table 1:** Lifetimes and associated states from global analysis of fs-TA spectra of three nitrophenolates in two solvents following excitation at two different wavelengths *.

Molecule	Exc. (nm)	Solvent	$\tau_{ESA,1}$ (ps)	$\tau_{TICT}$ (ps)	$\tau_{ESA,2}$ (ps)	$\tau_{HGSA,1}$ (ps)	$\tau_{HGSA,\;2}$ (ps)	$\tau_{long}$ (ns)
2NP^−^	400	Water		0.23	0.21	1.2		
	267		<0.3	0.5		1.1		1.6
		Methanol	<0.3	0.7		1.2	9.6	
4NP^−^	400	Water		0.21	0.21	1.4		
	267		<0.3	<0.3	0.5	1.4		1.3
		Methanol	<0.3	<0.3	0.5	1.5	8.1	
3NP^−^	400	Water			0.5	1.4		
	267				0.6			0.89
		Methanol	<0.3		1.3	5.7		

* Red and blue shades highlight the retrieved lifetimes of transient electronic states or species after 400 and 267 nm excitations, respectively. Only the 267 nm excitation data are shown for nitrophenolates in methanol. Blank boxes indicate the absence of corresponding states/lifetimes. Further details about the global analysis procedures can be found in the Supplementary Materials (Figure S2), corroborating the longer excited-state relaxation for the nitrophenolates after 267 nm compared to 400 nm excitation (Figure 2). For the nitrophenolates in methanol data, the nonzero background signal subtraction procedure (see details in Figure S5) enables a focus on the transient electronic dynamics tabulated here on the fs-to-ns timescales. The impact on the absence of an apparent long-time constant in methanol after 267 nm excitation can be found after Figure S2 in the Supplementary Materials.

**Table 2:** Probe-dependent fitting parameters of nitrophenolates in basic water, retrieved from Figure 3 *.

Molecule	Exc. (nm)	Region Center (nm)	$t_{0}$ (ps)	FWHM (ps)	$\tau_{1}$ (ps)	$\tau_{2}$ (ps)	$\tau_{3}$ (ps)	$\tau_{4}$ (ps)
2NP^−^	400	515	–0.01	0.11	0.3	0.3	0.3	1.7
		615	0.01	0.13	0.1	0.2	0.3	7.4
	267	515	0.41 ^†^	0.41 ^†^	0.1	0.2	1.3	100
		615	0.37 ^†^	0.41 ^†^	<0.1	0.1	0.7	1000
4NP^−^	400	515	0.07	0.17	<0.1	<0.1	0.7	5.2
		615	0.08	0.17	0.1	0.2	0.1	22
	267	515	0.44 ^†^	0.39 ^†^	<0.1	0.1	0.9	1400
		615	0.40 ^†^	0.43 ^†^	<0.1	0.1	0.8	1300
3NP^−^	400	515	–0.05	0.10	0.3	0.3	2.5	
		615	0.02	0.20	0.2	0.2	16	
	267	515	0.46 ^†^	0.48 ^†^	<0.1	0.9	3.5	2000
		615	0.31 ^†^	0.55 ^†^	0.3	1.4	85	1700

* The listed values from the probe-dependent TA data best fits roughly correspond to the features reported by global analysis (see Table 1 above); however, the convolution of features due to spectral overlap hinder the accuracy of such an analysis. Red and blue shades highlight the retrieved parameters of transient electronic states/species after 400 and 267 nm excitations, respectively, for three nitrophenolates in water. Blank boxes indicate the absence of corresponding time constants. The fitting parameters with more detailed values can be found in Table S1, along with the probe-dependent fitting parameters for three nitrophenolates in methanol after 267 nm excitation, for comparison. Five datasets across each complete TA measurement (see Figure S8, for example) were averaged and a least-squares fit was performed to yield the time constants in this table for a systematic comparison among three regioisomers of nitrophenolates under 400 and 267 nm excitations. ^†^ These fitted time zero ($t_{0}$) and full-width at half-maximum (FWHM) values are noticeably larger due to the delayed onset of the TA signal’s maximal intensity magnitude following 267 nm excitation versus 400 nm excitation (see Figure 2d–f versus Figure 2a–c). Accordingly, the blue traces consistently exhibit a delayed peak maximum on the sub-ps timescale versus the red trace in all the panels of Figure 3 for all three nitrophenolates in water. Note that the differences go beyond the variation in cross-correlation times: ~120 fs with 400 nm excitation (compressed) and <300 fs with 267 nm excitation (uncompressed). Due to sample dispersion, the cross correlation (and hence, the effective temporal resolution of the experiments) could be wavelength-dependent even for a compressed probe pulse; yet, the best-fit $t_{0}$ and FWHM values in this table around 515 and 615 nm probe wavelengths are largely similar. Therefore, the focus here is not the exact numerical values or profiles for the cross-correlation time across a broad spectral window, but the systematic fitting and comparative analysis between the electronic dynamics (e.g., onset and decay) as a function of two excitation wavelengths (i.e., 400 and 267 nm in this work).

**Table 3:** Lifetimes and associated states from global analysis of the nitrophenol fs-TA spectra in Figure 6.

Molecule	Solvent	$\tau_{ESA,1}$ (ps)	$\tau_{SE}$ (ps)	$\tau_{ESA,2}$ (ps)	$\tau_{HGSA,1}$ (ps)	$\tau_{HGSA,2}$ (ps) ^a^	$\tau_{long}$ (ps)
2NP	Water	<0.3	<0.3		1.1		900
	Methanol	0.3		5.5			390
4NP	Water	<0.3	<0.3		2.1	36	4300
	Methanol	0.3		12	37	1300	
3NP	Water	<0.3		0.8			140
	Methanol	<0.3		4.3	5.0		810

^a^ The assignment of these lifetimes is based on the spectral evolution from the electronic excited state to the ground state with the expected blueshift of the HGSA band and the relative transition oscillator strength of the HGSA versus a long-lived trapped state. Further discussions can be found after the Figure S6 caption in the Supplementary Materials.

## Data Availability

All data needed to evaluate the conclusions in the paper are present in the paper and the Supplementary Materials.

## Supplementary Materials

The following supporting information can be downloaded at: https://www.mdpi.com/article/10.3390/molecules28020601/s1, Figure S1: Steady-state electronic absorption spectroscopy of nitrophenols as a function of pH with the determination of pK_a_ values, and an appendix figure on the absorption spectroscopy of 3-nitrophenolate in three solvents; Figure S2: Global analysis of fs-TA spectra of nitrophenolates in solution after near-UV and UV excitations; Figure S3: Steady-state and time-resolved electronic spectra of catechol in aqueous solution; Figure S4: Probe-dependent TA data with least-squares fits of the nitrophenolates after 267 nm excitation in solution; Figure S5: TA spectra of nitrophenols and nitrophenolates in methanol at early and late time points following 267 nm excitation; Figure S6: Global analysis of fs-TA spectra of nitrophenols in solution after 267 nm excitation; Figure S7: Probe-dependent TA data with least-squares fits of the nitrophenols after 267 nm excitation in solution; Figure S8: TA spectral dynamics of 2NP in water for five datasets across the experiment; Figure S9: Computations for Raman spectra of three nitrophenolates in water; Figure S10: Computations for Raman spectra of three nitrophenols in water; Figure S11: Calculated key Raman modes for three nitrophenolates in water; Figure S12: Calculated key Raman modes for three nitrophenols in water; Table S1: Parameters for the probe-dependent fits of nitrophenolates in basic water and methanol after 267 nm excitation; Table S2: Parameters for the probe-dependent fits of nitrophenols in water and methanol after 267 nm excitation; Table S3: Calculated ground-state Raman and experimental FSRS peak frequencies with vibrational normal-mode assignments of 2-nitrophenolate (2NP^−^) in water; Table S4: Calculated ground-state Raman and experimental FSRS peak frequencies with vibrational normal-mode assignments of 3-nitrophenolate (3NP^−^) in water; Table S5: Calculated ground-state Raman and experimental FSRS peak frequencies with vibrational normal-mode assignments of 4-nitrophenolate (4NP^−^) in water; Table S6: Calculated ground-state Raman and experimental FSRS peak frequencies with vibrational normal-mode assignments of 2-nitrophenol (2NP) in water; Table S7: Calculated ground-state Raman and experimental FSRS peak frequencies with vibrational normal-mode assignments of 3-nitrophenol (3NP) in water; Table S8: Calculated ground-state Raman and experimental FSRS peak frequencies with vibrational normal-mode assignments of 4-nitrophenol (4NP) in water; and Supplementary References [107,108,109,110,111,112,113].

## Sample Availability

Samples of the compounds are available from the authors.
