# Supplementary material for: Ultrafast Spectroscopies of Nitrophenols and Nitrophenolates in Solution: From Electronic Dynamics and Vibrational Structures to Photochemical and Environmental Implications
Source: Molecules. 2023 Jan 6;28(2):601. doi: 10.3390/molecules28020601 (PMC9866910; doi:10.3390/molecules28020601)
Supplement: Supplementary file 1 [file molecules-28-00601-s001.zip › molecules-2051938-supplementary.pdf]

Supplementary

Ultrafast Spectroscopies of Nitrophenols and Nitrophenolates in Solution: From Electronic Dynamics and Vibrational Structures to Photochemical and Environmental Implications

Sullivan Bailey-Darland, Taylor D. Krueger and Chong Fang *,†

Department of Chemistry, Oregon State University, 153 Gilbert Hall, Corvallis, OR 97331, USA

* Correspondence: Chong.Fang@oregonstate.edu; Tel.: +1-541-737-6704

‡ Web: https://fanglab.oregonstate.edu/.

Table of Contents

1. Supplementary Figures

Figure S1. Steady-state electronic absorption spectroscopy of nitrophenols as a function of pH with the determination of pK_a values (with appendix figure)...... S3

Figure S2. Global analysis of fs-TA spectra of nitrophenolates in solution after near-UV and UV excitations …………………………………………………..…. S5

Figure S3. Steady-state and time-resolved electronic spectra of catechol in aqueous solution ……………………………………………………………….... S8

Figure S4. Probe-dependent TA data with least-squares fits of the nitrophenolates after 267 nm excitation in solution ………………………………………….………. S9

Figure S5. TA spectra of nitrophenols and nitrophenolates in methanol at early and late time points following 267 nm excitation …………………...…….….… S10

Figure S6. Global analysis of fs-TA spectra of nitrophenols in solution after 267 nm excitation ……………………………………………………...…….….… S11

Figure S7. Probe-dependent TA data with least-squares fits of the nitrophenols after 267 nm excitation in solution ……………………………………….....… S14

Figure S8. TA dynamics of 2NP in water for five experimental datasets…....…. S15

Figure S9. Calculated Raman spectra of three nitrophenolates in water …….... S16

Figure S10. Calculated Raman spectra of three nitrophenols in water ………...S17

Figure S11. Calculated key Raman modes for nitrophenolates in water ……..... S18

Figure S12. Calculated key Raman modes for nitrophenols in water ……….….S18

2. Supplementary Tables

Table S1. Parameters for the probe-dependent fits of nitrophenolates in basic water and methanol after 267 nm excitation ………………………………….. S19

Table S2. Parameters for the probe-dependent fits of nitrophenols in water and methanol after 267 nm excitation ………………………………………..…. S21
Table S3. Calculated ground state Raman and experimental FSRS peak frequencies with mode assignments of $2NP^-$ in water ………………...….… S23
Table S4. Calculated ground state Raman and experimental FSRS peak frequencies with mode assignments of $3NP^-$ in water ………………...….… S24
Table S5. Calculated ground state Raman and experimental FSRS peak frequencies with mode assignments of $4NP^-$ in water ………………...….… S25
Table S6. Calculated ground state Raman and experimental FSRS peak frequencies with mode assignments of 2NP in water …………………………. S26
Table S7. Calculated ground state Raman and experimental FSRS peak frequencies with mode assignments of 3NP in water …………………………. S27
Table S8. Calculated ground state Raman and experimental FSRS peak frequencies with mode assignments of 4NP in water …………………………. S28

3. Supplementary References…………………………….………………… S29

1. Supplementary Figures

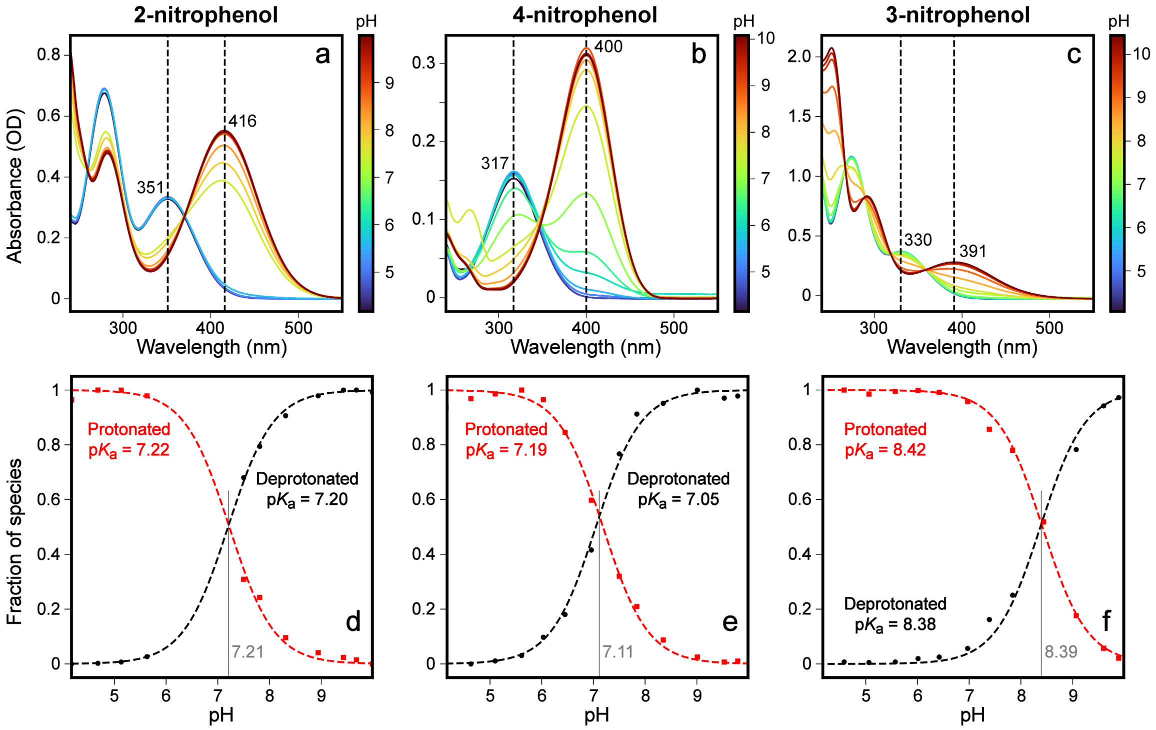

Figure S1. Steady-state electronic absorption spectroscopy of nitrophenols as a function of pH with the determination of pK_a values. (**a-c**) UV/Visible absorption spectra of the nitrophenols at varying pHs, where the pH of each line is indicated by the color bar on the right side. Characteristic wavelengths used to determine the molar fraction of the protonated and deprotonated species are indicated by vertical dashed lines, labeled with the peak wavelength. (**d-f**) Calculated molar fraction of each species at varying pHs. Measured data points (red squares and black dots) are overlaid with the best fits (color-coded dashed lines) for the protonated (red) and deprotonated (black) species, respectively. The pK_a of each fit is shown, and the average of both fits in each panel is largely consistent (within 0.08) with the intersection of the fits (vertical gray line, the corresponding pK_a value is labeled in each panel).

We note that the pK_a values of 2/4NP are lower than 3NP, which likely originates from the *meta*-effect in 3NP that decreases its acidity at the phenolic hydroxy group in S_0 at thermal equilibrium. The altered electron density maps [1,2] involve resonance and inductive effects, which could underlie the increased tendency for 2NP and 4NP to undergo excited-state intramolecular proton transfer (ESIPT) and excited-state proton transfer (ESPT), respectively.

Moreover, we used steady-state electronic absorption spectroscopy to perform a control experiment on 3-nitrophenolate ($3NP^-$) in basic water (pH≈10) and to examine its potential solvatochromic properties. Besides the significant peak wavelength redshift for $3NP^-$ in acetonitrile versus water (see Section 2.1 in main text) as shown by the green and blue traces in the Appendix figure below, there is an apparent ~3 nm blueshift of the absorption peak in methanol versus water, which could inspire future studies on the solvatochromism of the nitrophenol and nitrophenolates across a wider range of solvents with diverse properties.

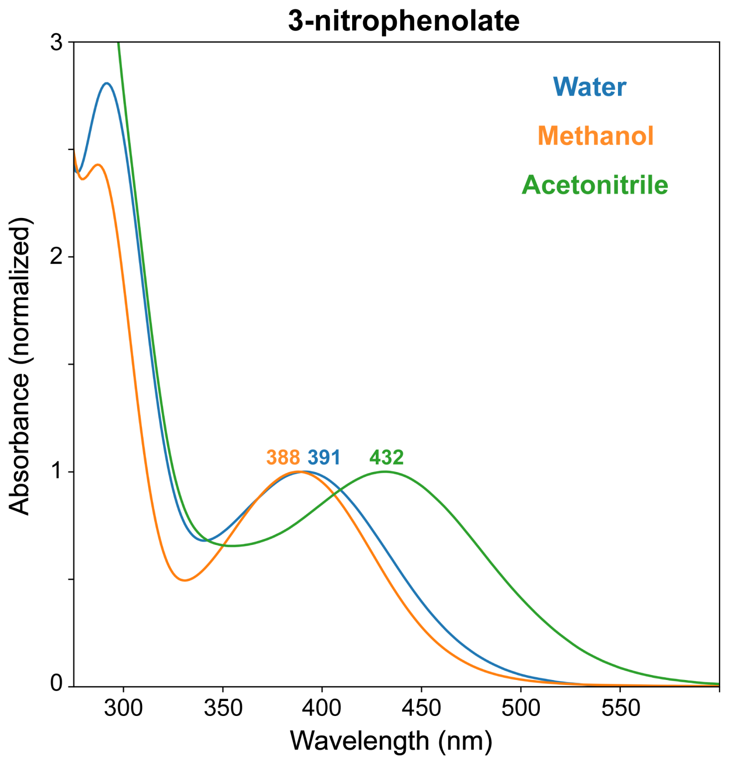

Figure S1 appendix. Steady-state absorption spectroscopy of 3-nitrophenolate in three solvents under basic conditions (see Section 4.1 in main text for sample solution preparation) at room temperature. The $3NP^-$ absorption spectra in water (blue), methanol (orange), and acetonitrile (green) are normalized at the reddest peak for a better comparison, with the color-coded peak wavelengths labeled. The significant peak redshift upon the solvent change from water (391 nm) to the least polar acetonitrile (432 nm) is notable.

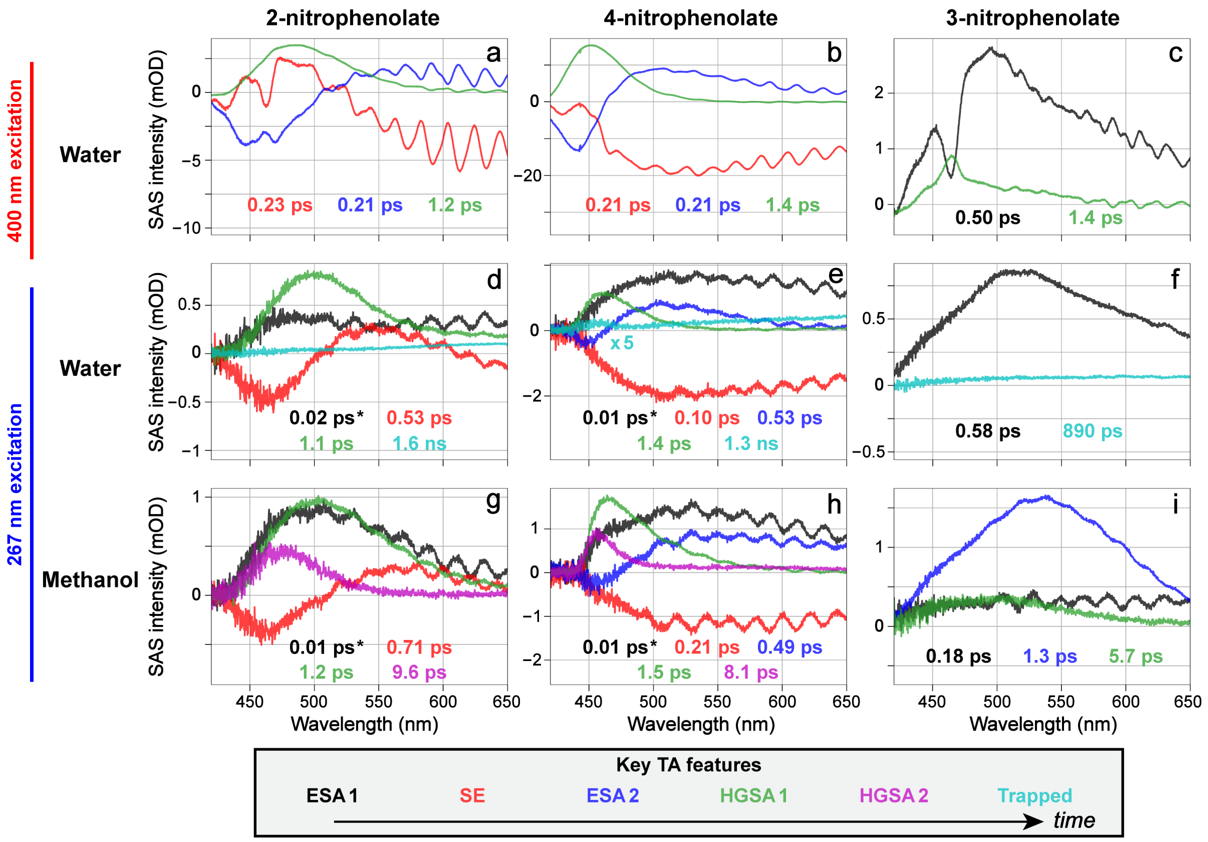

Figure S2. Global analysis of fs-TA spectra of nitrophenolates in solution after near-UV and UV excitations. Spectral components of (**a**) 2-nitrophenolate/2NP$^-$, (**b**) 4-nitrophenolate/4NP$^-$, (**c**) 3-nitrophenolate/3NP$^-$ after 400 nm excitation in water, (**d**) 2NP$^-$, (**e**) 4NP$^-$, and (**f**) 3NP$^-$ after 267 nm excitation in water, and (**g**) 2NP$^-$, (**h**) 4NP$^-$, and (**i**) 3NP$^-$ after 267 nm excitation in methanol. Each trace corresponds to the spectral profile of a particular state/species in the sequential model, with the color-coded lifetimes of the respective profiles shown in the insets. The color roughly corresponds to general features (listed in the bottom Key) observed in the experimental TA data. The asterisks (*) in panels **d**, **e**, **g**, and **h** denote the early-time transient electronic features that are contaminated by coherent artefacts (also present in other panels but with less effects), leading to minuscule lifetimes well within the instrument response function.

Notably, global analysis can fit most of the spectral features [3], though early-time features within the cross-correlation time after 267 nm excitation cannot be satisfactorily resolved. The retrieved lifetimes represent best fits of dynamic spectral profiles (see Table 1 in main text) as reported by Glotaran [4]. In particular, the best-fit spectral components in Figure S2d,e,g,h include an initial broad excited-state absorption (ESA) band with a minuscule lifetime of ~10–

20 fs, while a broad negative band such as ground-state bleaching (GSB) or stimulated emission (SE) follows due to the evolving spectral pattern as the molecule departs from Franck-Condon region [5]. These early-time spectral components may be heavily overlapped to hinder a quantitative determination of individual lifetimes, but the spectral profile and peak position retrieved are still representative of key relaxation pathways [3,6], such as a twisted intramolecular charge transfer (TICT) state with a red-shifted SE band of 2/4NP^{-} that favors nitro-group twisting in the excited state (e.g., Figure S2a,b,d,e in water). The blue negative feature could be an SE band from the planar fluorescent state. Later features are reported more accurately, since the cross-correlation time of ~100–300 fs across near-UV to UVC range in the setup is much shorter than the lifetime of transient electronic state(s). Both HGSA1 and HGSA2 components report the vibrational relaxation process in the electronic ground state from the transiently "hot" ground state toward the equilibrated "cold" ground state. All the displayed spectral components were scaled according to the respective amplitude weights from best fits in global analysis, and verified to recover the original TA spectra (see Section 4.6).

Interestingly, a "trapped state" shows up for nitrophenolates after 267 nm excitation in water (Figure S2d,e,f), which generally has a broad red absorption band (>550 nm) and a long lifetime (>900 ps). This feature can be fit by global analysis with a lifetime over 1 ns, while the length of our detection time window (900 ps) can support a rather accurate determination of a few ns time constant due to the nature of an exponential decay function [7]. A more exact lifetime determination would require a longer measurement window, and can be the subject of future work with a focus on intersystem crossing (ISC) and potential triplet state dynamics on much longer timescales (e.g., tens of ns to μs). A close inspection shows that the ns component for 2/4/3NP^{-} in water (Figure S2d,e,f) exhibits an absorption band redder than 550 nm (with a peak redder than ~600 nm), whereas the longest component for 2/4/3NP^{-} in methanol (Figure S2g,h,i) has an absorption peak bluer than ~500 nm. This spectral difference could be affected by the subtraction of a nonzero background signal (a broad absorption band with a ~570 nm peak) in methanol, but the pertinent long-lived state shows discernible differences versus water.

To examine the effect of nonzero background subtraction (see Figure S5 below) on the global analysis of TA data, we also retrieved the lifetimes without the spectral subtraction but with the same kinetic model in global analysis. For 2NP^{-} in methanol (Figure S2g: 0.01, 0.71,

1.2, and 9.6 ps), the retrieved lifetimes become 0.1, 0.78, 2.6, and 2500 ps; for $4NP^-$ in methanol (Figure S2h: 0.01, 0.21, 0.49, 1.5, and 8.1 ps), the retrieved lifetimes become 0.01, 0.24, 0.61, 3.6, and 2100 ps; and for $3NP^-$ in methanol (Figure S2i: 0.18, 1.3, and 5.7 ps), the retrieved lifetimes become 0.43, 2.5, and 4600 ps. Therefore, the major difference lies in the long-time constant that extends to the ns timescale which is expected as the nonzero background TA signal was kept throughout the detection window (i.e., masking the light-induced TA spectral evolution that starts from the time zero). The focus on TA dynamics due to actinic pump (i.e., photoexcitation) is thus warranted as the systematic comparisons between three nitrophenolates (Figures 2 and S2), particularly the HGSA decay constants on the ps timescale (e.g., τ_{HGSA} and $\tau_{HGSA,2}$ in Figure S2 above and Table 1 in main text), instead of the lingering processes that likely involves a long-lived triplet or dark state while exposing a stagnant positive band before the actinic pump hits the sample at time zero (see further discussions below, and the footnote of Table 1 in main text). It is also evident from the aforementioned numerical comparisons that the early-time constants/lifetimes are largely insensitive to the subtraction of this stagnant positive band from the nitrophenolate TA spectra (Figure 2g-i and Figure S2g-i) in methanol after 267 nm excitation.

Furthermore, fs-TA spectra after 400 nm excitation require a fast early feature to fit the strong coherent artefact (see the "Materials and Methods" Section 4.6 on "Global Analysis" in main text). This result was due to high temporal compression and short pulse duration of the 400 nm laser, especially compared to the 267 nm experiments. These features were fit by fixing an ultrafast lifetime (0.01 ps) to fit only the coherent artefact. In addition, removing the feature did not significantly affect the fitting of later states/features, which may show some slight distortions of the absorption profiles as reported in Figure S2 but the lifetimes are largely unchanged. Therefore, we mitigated the issue by a systematic comparison between various samples under a series of experimental conditions so a consistent dynamic picture can be obtained to expose spectral similarities and differences. For $3NP^-$, the coherent artefact required two features to fit, and these were fixed at 0.01 and 0.02 ps, respectively, before subtraction to yield the dynamic components of interest (Figure S2c).

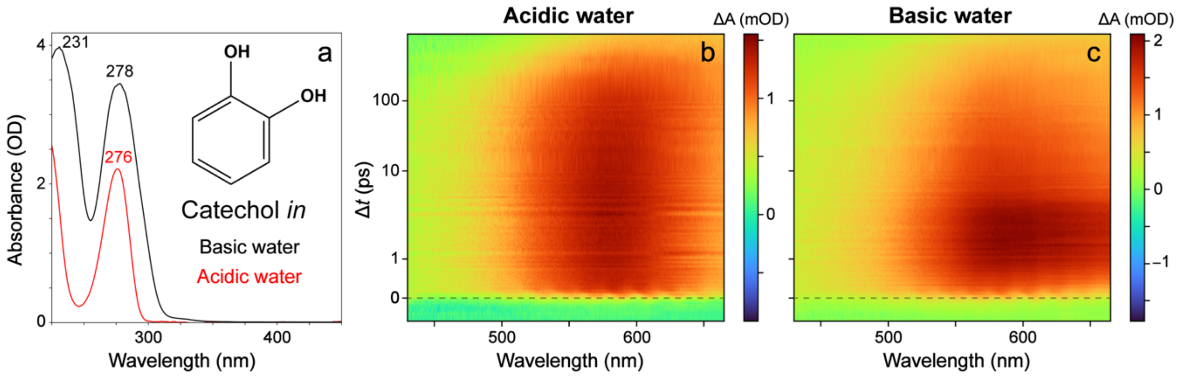

Figure S3. Steady-state and time-resolved electronic spectra of catechol in aqueous solution. (**a**) UV/Vis spectra of catechol in basic (black) and acidic (red) water show some subtle changes, with peak locations denoted. The chemical structure of catechol is shown in the inset. Fs-TA spectra of catechol in (**b**) acidic water (pH≈4) and (**c**) basic water (pH≈10) after 267 nm excitation exhibit a promptly emerged ESA band around time zero (denoted by dashed lines).

The pK_a values of catechol were reported to be ~9.3 and 13 [8,9], so at pH = 10 an inhomogeneous mixture of catechol is expected. After UVC-light irradiation, catechol in water displays a drastically increased excited-state lifetime compared to 2NP (see Figure 1 inset), where the molecules only differ by the functional group that is *ortho* to the phenolic hydroxy group (regardless of its protonation state). In 2NP, the nitro-group twist (and some pyramidalization) allows efficient relaxation, and excited-state TA features mostly disappear on the ps timescale (Figure 2). In catechol, no such twist can occur, so the excited-state lifetime is substantially elongated to hundreds of ps and beyond, reminiscent of recent TA results of nonaggregated catechol in acetonitrile with ~710 ps lifetime (S_1 state) [10].

Furthermore, it is worth noting that the fs-TA measurements of catechol in acidic water (see Figure S3b above) were taken on the same day, using the same experimental setup for data collection on the nitrophenolates in water (with 267 nm excitation, see Figure 2d,e,f in main text). All the nitrophenolate samples (2/3/4NP$^-$) demonstrate a delayed rise in the prominent ESA band (see blue traces in Figure 3 for better illustration), which may be due to an inaccurately calibrated time zero or intrinsic molecular dynamics at early times with weak absorption or emission in the spectral window. Since the catechol fs-TA data from the same day do not show this delayed onset of ESA band after the same UVC (267 nm) excitation, we can more confidently attribute the delay in the nitrophenolates to real molecular dynamics.

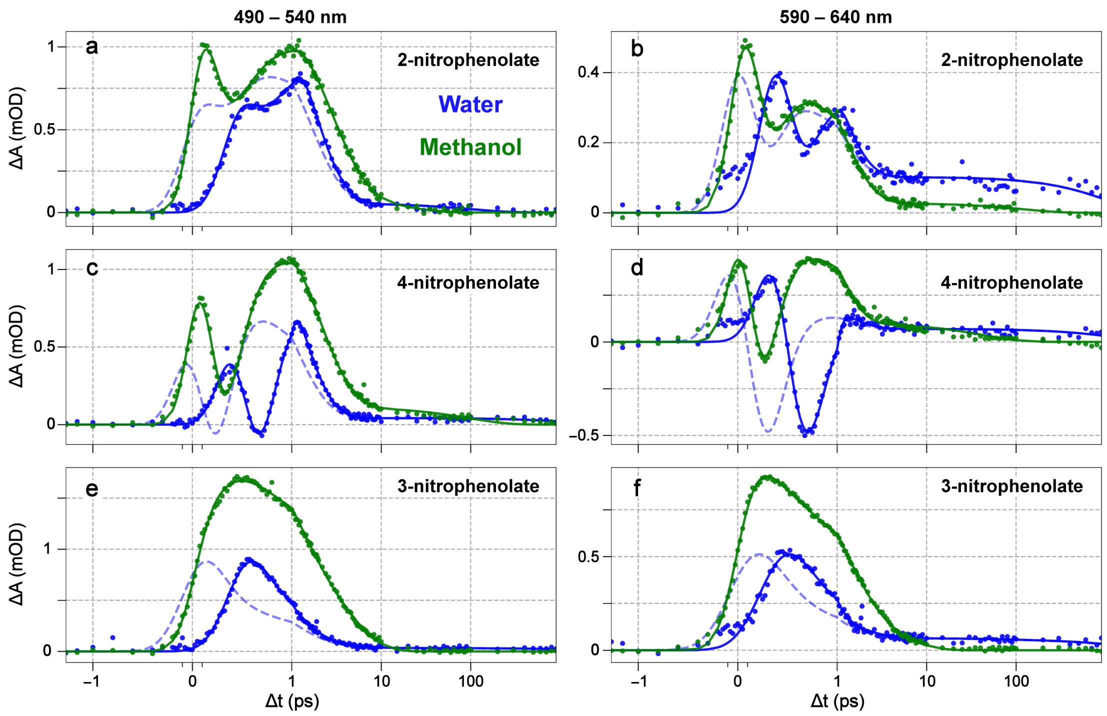

Figure S4. Probe-dependent TA data with least-squares fits of the nitrophenolates after 267 nm excitation in solution. Spectral data intensities are integrated and averaged within the regions of 490–540 nm (left) and 590–640 nm (right) for (**a**, **b**) 2-nitrophenolate, (**c**, **d**) 4-nitrophenolate, and (**e**, **f**) 3-nitrophenolate in water (blue) and methanol (green), respectively. Spectral data points (filled dots) are overlaid with the best fits (color-coded solid lines). The early-time small absorption band visible at longer wavelengths (right column) in water is not fit, which results in a delayed time zero. To better compare the methanol and water data in each panel, the fit for the water data is shifted by the fitted time-zero offset and plotted as a dashed blue line. Moreover, to account for the non-zero background in the methanol data, the average of the first five time points (pre-excitation) was subtracted from the TA data for analysis (Figure S5).

Notably, the temporally shifted (toward early time, by a few hundreds of fs) TA data plots for all three nitrophenolates in water show a much improved resemblance to the TA data plots in methanol, indicating that the 267 nm light-induced initial access to higher-lying electronic states (e.g., S_n) is more prominent in water that shows a redder ESA band (e.g., $S_n \rightarrow S_{n+1}$) than the later appeared, prominent ESA band from a relaxed electronic state (e.g., $S_1' \rightarrow S_2$, the prime distinguishes the electronic state from S_1 upon 400 nm excitation). The prominent ESA and SE features are thus mostly associated with the lower-lying S_1 or S_1' state (Figure 2d-i, main text), while the ESA and ensuing HGSA features become more apparent as the SE band decays away.

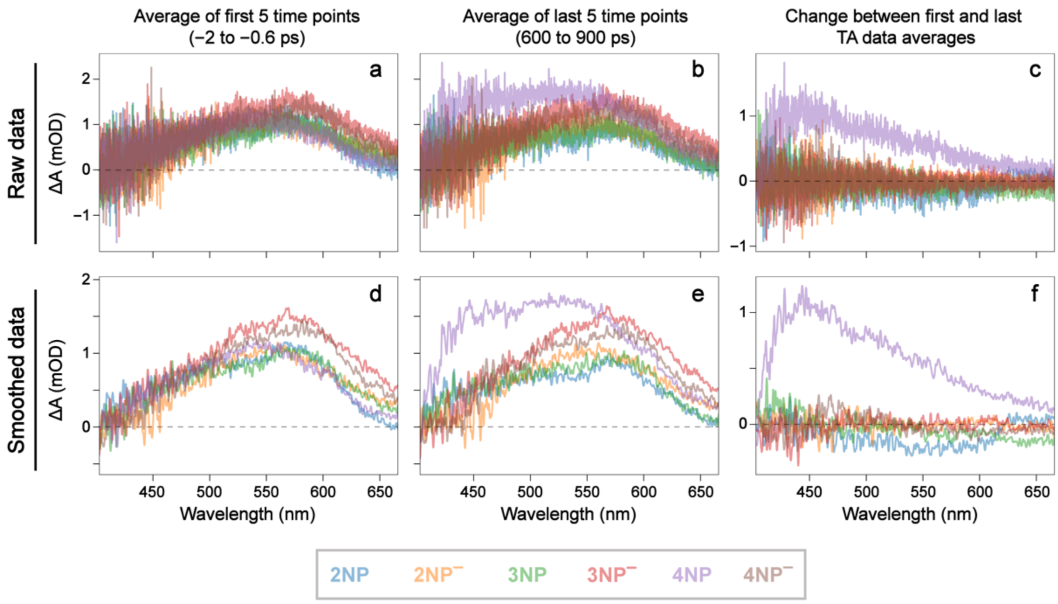

Figure S5. TA spectra of nitrophenols and nitrophenolates in methanol at early and late time points following 267 nm excitation. Raw experimental spectra for (**a**) the average of first five time points (i.e., pre-excitation), (**b**) the average of last five time points (i.e., post-excitation), and (**c**) the change between first and last averaged data (i.e., **b** – **a**) are shown in the top panels, while the corresponding smoothed spectra (using a 1D Gaussian filter) are displayed in (**d**), (**e**), and (**f**) in bottom panels for 2NP (cyan), $2NP^-$ (orange), 3NP (green), $3NP^-$ (red), 4NP (purple), and $4NP^-$ (brown), respectively. The key for each color-coded molecule is listed in the rectangular box below. The zero absorbance is denoted by a gray dashed line in each panel.

Notably, TA measurements of all the nitrophenols and nitrophenolates in methanol exhibit an unusual pre-excitation absorption (Figure S5a,d), resembling the post-excitation absorption at the end of detection time window (Figure S5b,e). Subtracting these two features shows little spectral change across the detection spectral window (Figure S5c,f), except for 4NP that has a significant long-lasting absorption band (see purple traces in Figure S5c,f, attributed to HGSA, Figure S6). Due to experimental data collection with a 1 kHz laser repetition rate, the "residual" absorption would have to last on the order of milliseconds to remain from one excitation to the next measurement window, which could be due to some long-lived "trapped state" in methanol. Since this background signal exhibits little variation throughout the detection window as confirmed by Figure S5c, the aforementioned spectral subtraction was performed for all the TA data analysis for samples in methanol to highlight the transient electronic dynamics of interest.

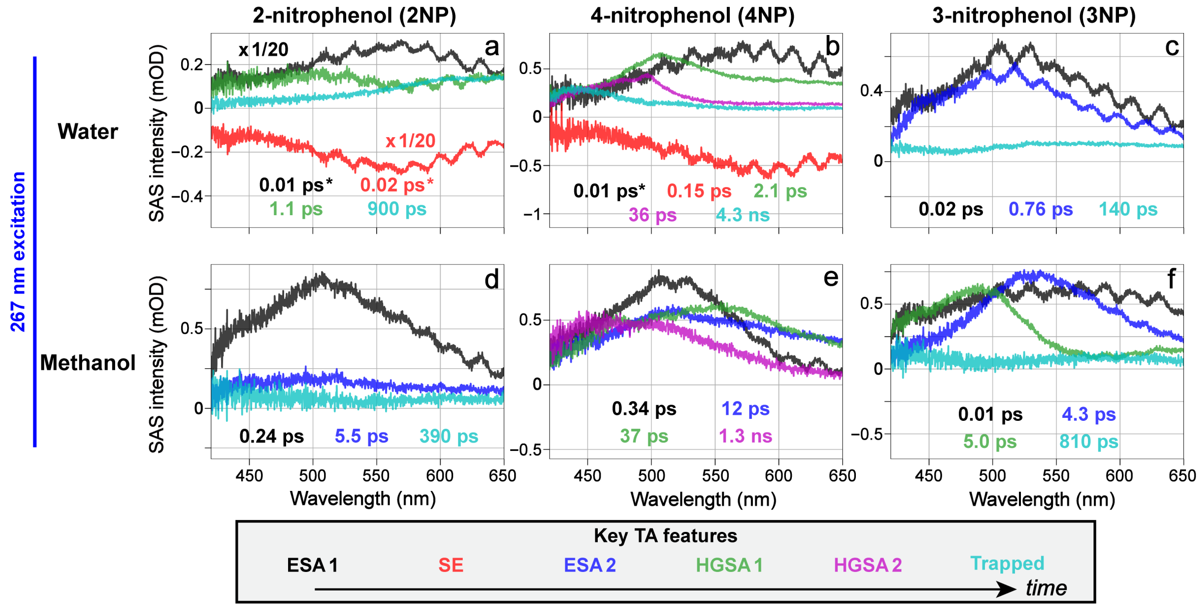

Figure S6. Global analysis of fs-TA spectra of nitrophenols in solution after 267 nm excitation. The UV-light-induced transient spectral components are shown for (**a**) 2-nitrophenol/2NP, (**b**) 4-nitrophenol/4NP, (**c**) 3-nitrophenol/3NP in water, and (**d**) 2NP, (**e**) 4NP, and (**f**) 3NP in methanol. Each trace corresponds to the spectral profile to a particular state/species in the sequential model, with the color-coded lifetimes and scaling factors (if applied) shown. The color roughly corresponds to general features (listed in the bottom Key) observed in the TA data. The asterisks (*) in panels **a** and **b** denote early-time transient electronic features that are contaminated by coherent artefacts and thus fixed during global analysis (see detailed methods in Section 4.6), leading to minuscule lifetimes well within the instrument response function.

Global analysis was able to fit the spectra well, with the most complicated spectra being 2NP and 4NP in water (Figure S6a,b). Rich spectral features may arise from the ES(I)PT capability of 2/4NP in water, in accord with a red SE band at early times (see Figure S6a,b and Figure 6a,b in main text), whereas such processes are inhibited in methanol as previously observed for another photosensitive chromophore called pyranine [11,12]. In the case of 2NP, the retrieved components from global analysis was complicated by the low signal strength and some sample degradation that occurred during the measurement (see Figure S8 below, and Figure 7b in main text). These spectra required the first feature to have a fixed ultrafast time constant. The exact value is likely unimportant, and we tried lifetimes from 1–200 fs that did

not affect the fitting of later features. A representative value of 10 fs was then selected. For 2NP, likely owing to its lower TA signal strength, the second feature needed to be fixed as well, and 20 fs was used. Though the second feature appears almost as the negative "mirror image" of the first feature, there are some noticeable differences (from peak positions to intensity ratios). Due to contamination by coherent artefacts around time zero with a larger cross-correlation time using 267 nm pump (see fs-TA spectroscopy "Methods" Section 4.3 in main text), we did not consider these components as an accurate representation of the early-time electronic dynamics of 2/4NP in water, and hence did not include these numbers in Table 3 as the pertinent nitrophenol electronic dynamics are discussed. However, the probe-dependent fits corroborate the occurrence of a weak SE band within the spectral regions of 490–540 and 590–640 nm (Figure S7, especially in water), consistent with the spectral profile of second component from global analysis (see Figure S6a,b, red trace assigned to SE band in the Key). Moreover, an SE band from TICT state is generally broad and red-shifted from other TA features [13-15], in accord with the swift nitro-group twisting on ultrafast timescales en route to a sloped S_1/S_0 conical intersection in 2/4NP (see a visual illustration for the relevant 2/4NP$^-$ in Figure 5a, main text), both in water and methanol.

Interpreting the subsequent global analysis features of the three nitrophenols is relatively straightforward, mainly tracking the excited-state relaxation pathways via a decaying broad absorption band. One exception is the 1.3 ns feature found for 4NP in methanol, which is labeled as a HGSA contribution (Figure S6e, magenta trace; unlikely to be attributed to ESA due to its ns lifetime for an essentially nonfluorescent molecule — we could not detect its fluorescence peak), also evident from the background-subtracted TA signal (see Figure S5c,f above). This assignment is based on the unusually large magnitude of this specific spectral component with a clear blueshift from the preceding feature (Figure S6e, green trace, assigned to HGSA1 in the Key) and a clearly delayed onset (Figure S7c,d, green traces), as evident from the 2D-contour plot of TA spectra (Figure 6e in main text). A long-lived trapped state may still exist, but its associated weak spectral feature (as suggested by the cyan trace in Figure S6d) is likely diminished through the background signal subtraction process (Figure S5c,f), and the remaining spectral feature is clearly overwhelmed by the much stronger HGSA band that has a characteristic lifetime of ~1.3 ns (Figure S6e, magenta trace, assigned to HGSA2 in the Key).

In addition, the significantly longer lifetime of this HGSA2 spectral feature of 4NP in methanol after 267 nm excitation than the counterparts of 2/3/NP (Figure S6d,f, also see Table 3 in main text) is indicative of an energy barrier in the ground state that 4NP needs to overcome on the ns timescale to return to the thermally equilibrated S_0 state [14,16-18].

To examine the effect of nonzero background subtraction (Figure S5) on the global analysis of TA data, we also obtained the lifetimes without the spectral subtraction but with the same kinetic model. For 2NP in methanol (Figure S6d: 0.24, 5.5, and 390 ps), the retrieved lifetimes become 0.28, 3.8, and 930 ps; for 4NP in methanol (Figure S6e: 0.34, 12, 37 ps, and 1.3 ns), the retrieved lifetimes become 0.32, 14, 31 ps, and 1.7 ns; and for 3NP in methanol (Figure S6f: 0.01, 4.3, 5.0, and 810 ps), the retrieved lifetimes become 0.01, 4.4, 4.9, and 1100 ps. Therefore, the major difference lies in the long-time constant on the hundreds of ps to a few ns timescale, which is expected as the nonzero background TA signal was kept throughout the detection window. The focus on the transient TA dynamics due to actinic pump (i.e., photoexcitation) is thus warranted as the systematic comparisons between nitrophenolate (Figures 2 and S2) or nitrophenol (Figures 6 and S6) samples should yield crucial insights into the excited-state electronic dynamics of the chromophore in solution with rapidly perturbed solute-solvent interactions starting from time zero, instead of the lingering processes that may involve a long-lived triplet or dark state [5,14,19].

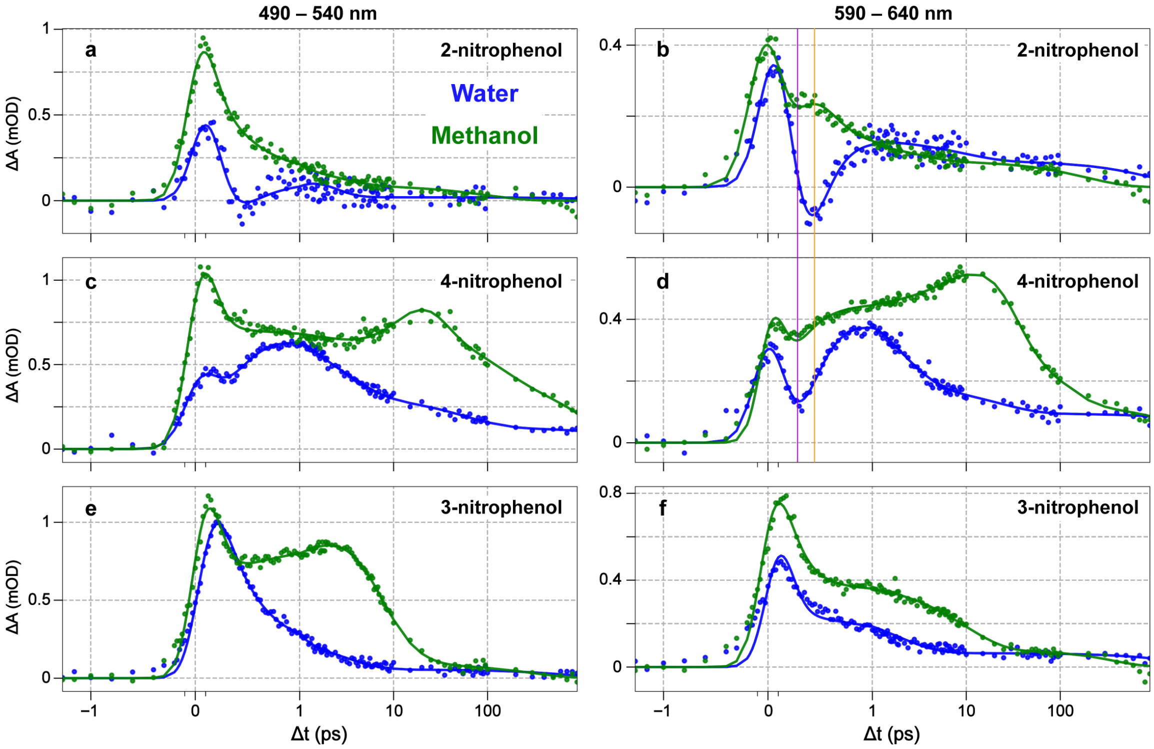

Figure S7. Probe-dependent TA data with least-squares fits of the nitrophenols after 267 nm excitation in solution. Spectral data intensities are integrated and averaged within the regions of 490–540 nm (left) and 590–640 nm (right) for (**a**, **b**) 2NP, (**c**, **d**) 4NP, and (**e**, **f**) 3NP in water (blue) and methanol (green), respectively. Spectral data points (filled dots) are overlaid with the best fits (color-coded solid lines). To account for the non-zero background in the methanol data, the average of the first five time points (pre-excitation) was subtracted from the TA data across the detection time window for analysis (see Figure S5 above). Two vertical lines across panels **b** and **d** highlight the similar temporal position of the dips for 2/4NP in methanol (green traces, crossed by the vertical magenta line), a slightly delayed dip for 4NP in water (panel **d**, blue trace), and a notably delayed dip for 2NP in water (panel **b**, blue trace, crossed by the vertical orange line).

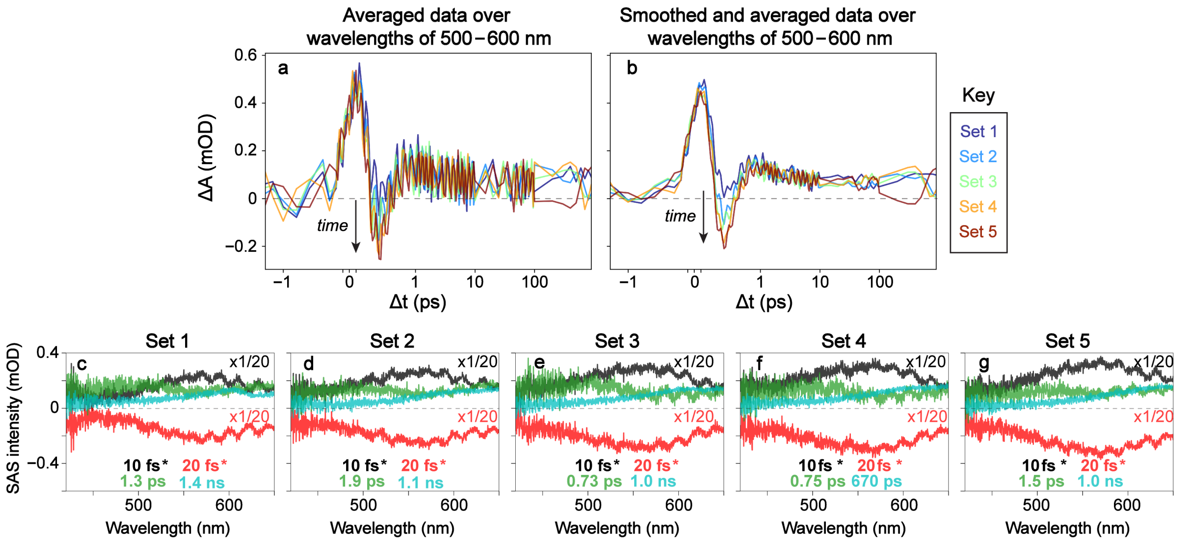

Figure S8. TA spectral dynamics of 2NP in water for five datasets across the experiment. The spectral data intensities are integrated and averaged within the region of 500–600 nm for (**a**) raw data and (**b**) smoothed data after applying a 1D Gaussian filter. Each dataset is color-coded to the Key on the right side. (**c**, **d**, **e**, **f**, **g**) Global analysis of each dataset, respectively, with the first two time constants fixed to fit the early-time dynamics with coherent artefacts (denoted by black and red asterisks, see Section 4.6). There is no significant change in the features or lifetimes across five sets, apart from varying lifetimes for the third (green) trace (0.7 to 1.9 ps).

Notably, TA dynamics in the spectral region of interest (Figure S8a,b) do not change much between sequential datasets, but the spectral intensities change. More significantly, the negative SE band grows stronger with time (see the downward arrows). Since 2NP degraded during 267 nm fs-TA measurements in water (Figure 7b), each dataset (still tracking the photoresponse of non-degraded subpopulations) was analyzed to examine if the usual procedure of average dynamics over all five datasets (to improve signal-to-noise ratio) is appropriate. No significant lifetime change was found, particularly about locations of the maxima and minima in the probe-dependent plots (Figure S8a,b). There is some signal intensity change: the SE feature at ~200 fs grows. Since 2NP signal is already weak (due to low sample solubility, transition oscillator strength, photoproduct spectral features outside the current detection window, etc.), the average of datasets was deemed beneficial to achieve better signal and more important than an accurate determination of the relative strength of SE feature. Therefore, TA data for 2NP after 267 nm excitation in water (see Figure 6a in main text) are shown with the average over all five datasets.

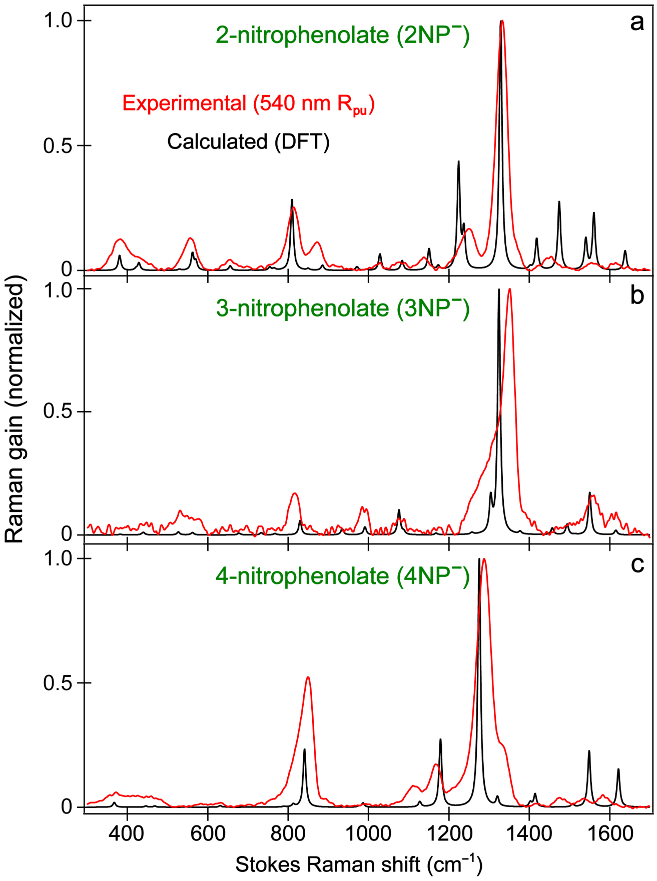

Figure S9. Computations for Raman spectra of three nitrophenolates in water. The experimental ground-state FSRS spectra (red traces) with 540 nm Raman pump (R_{pu}) and a redder Raman probe on the Stokes side for (**a**) $2NP^-$, (**b**) $3NP^-$, and (**c**) $4NP^-$ are overlaid with the calculated spectra using DFT (black traces, see Section 4.4 for detailed methods). Main peak intensities are normalized (between 1200–1400 cm^{-1}) for a better comparison between the two spectra in each panel.

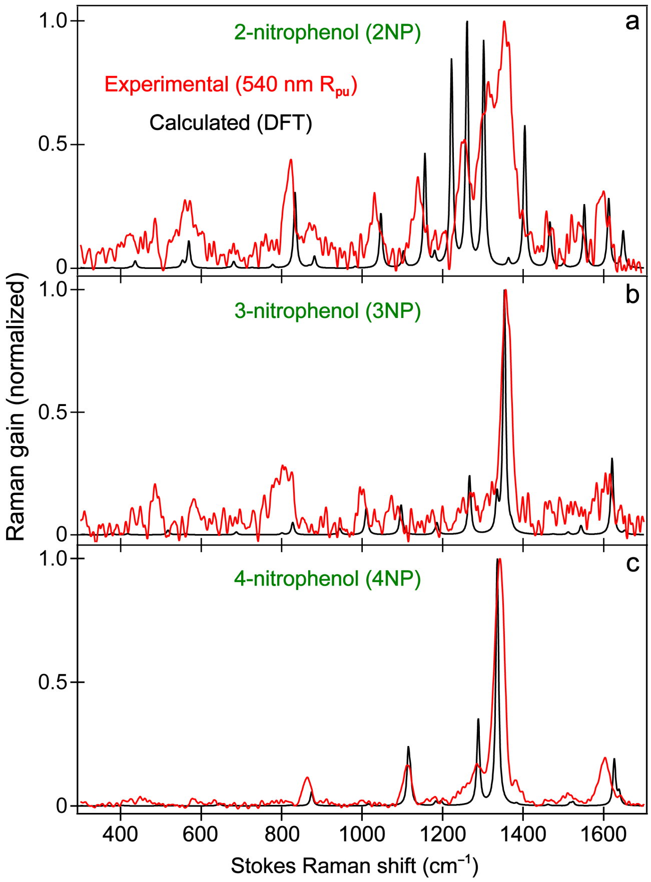

Figure S10. Computations for Raman spectra of three nitrophenols in water. The experimental ground-state FSRS spectra (red traces) with 540 nm Raman pump (R_{pu}) and a redder Raman probe on the Stokes side for (**a**) 2NP, (**b**) 3NP, and (**c**) 4NP are overlaid with the calculated spectra using DFT (black traces, see Section 4.4 for detailed methods). Main peak intensities are normalized (between 1200–1400 cm^{-1}) for a better comparison between the two spectra in each panel.

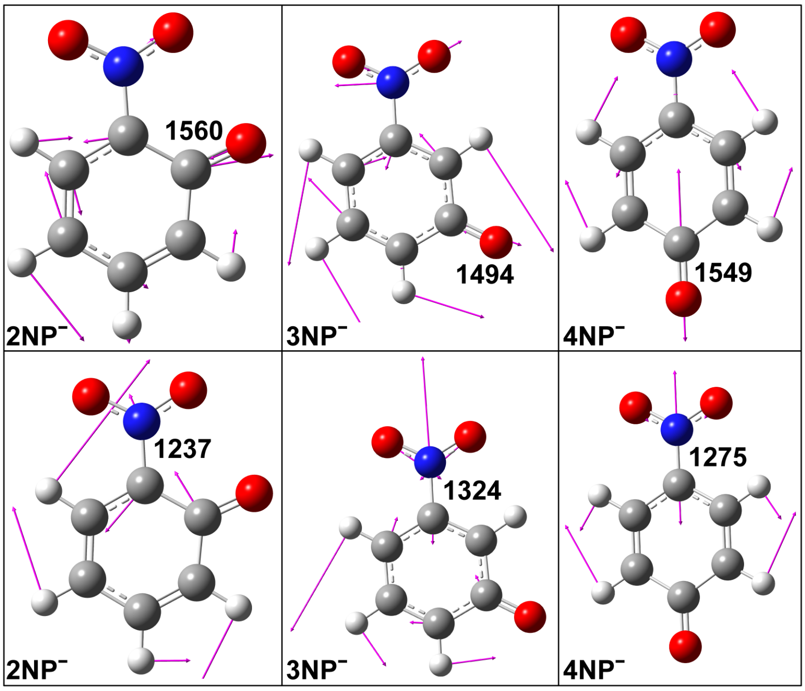

Figure S11. Calculated key Raman modes for three nitrophenolates in water. The primary vibrational normal modes for the C=O stretch at ~1560 cm^{-1} ($2NP^{-}$), 1494 cm^{-1} ($3NP^{-}$), and 1549 cm^{-1} ($4NP^{-}$) are illustrated in the top panels, with the C–N stretch at ~1237 cm^{-1} ($2NP^{-}$), 1324 cm^{-1} ($3NP^{-}$), and 1275 cm^{-1} ($4NP^{-}$) illustrated in the bottom panels. The pertinent atomic displacements are depicted by magenta arrows.

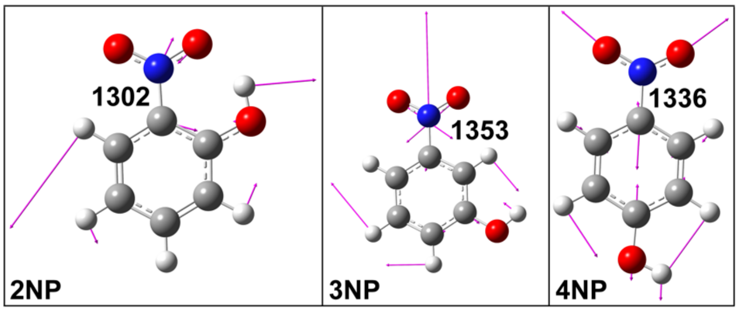

Figure S12. Calculated key Raman modes for three nitrophenols in water. The primary vibrational normal modes for the C–N stretch at ~1302 cm^{-1} (2NP), 1353 cm^{-1} (3NP), and 1336 cm^{-1} (4NP) are illustrated from left to right panels. The pertinent atomic displacements are depicted by magenta arrows. Note that a prominent C=O stretching mode is not present in these protonated chromophores (i.e., with the phenolic hydroxy group).

2. Supplementary Tables

Table S1. Parameters[a] for the probe-dependent fits of nitrophenolates in basic water and methanol after 267 nm excitation (TA data plots shown in Figure S4 above).

Molecule	Solvent	Region center (nm)	t_0 (ps)[b]	FWHM (ps)	τ_1 (ps)	τ_2 (ps)	τ_3 (ps)	τ_4 (ns)[c]
$2NP^-$	Water	515	0.41	0.41	0.12	0.18	1.3	0.1
		615	0.37	0.41	0.06	0.10	0.73	1.0
	Methanol	515	0.05	0.30	0.10	0.33	2.5	0.016
		615	0.03	0.34	0.09	0.14	0.87	0.12
$4NP^-$	Water	515	0.44	0.39	0.06	0.10	0.92	1.4
		615	0.40	0.43	0.07	0.10	0.83	1.3
	Methanol	515	0.06	0.31	0.07	0.22	1.7	0.094
		615	0.03	0.31	0.04	0.23	0.54	0.042
$3NP^-$	Water	515	0.46	0.48	0.08	0.87	3.5	2.0
		615	0.31	0.55	0.27	1.4	85	1.7
	Methanol	515	0.04	0.36	0.04	0.21	0.76	0.0035
		615	0.01	0.38	0.08	0.13	1.1	0.0042

[a] Values are reported to 2 significant figures (sig. fig.) or to the nearest 10 fs (whichever is more restrictive). The "Region center" column is the center wavelength in nm unit for the TA signal to be integrated and averaged for the least-squares fitting analysis. Most of the lifetimes are not as accurate as shown here, but are reported here exactly as found from the best fitting for model transparency. Each probe-dependent data set, fit with a sequential model, required only four components to fit well. Explanations of the parameters can be found in the Methods (Section 4.5, main text). Blue and green shades highlight the retrieved parameters from fs-TA spectra of the (deprotonated) nitrophenolate samples after 267 nm excitation in water and methanol, respectively. The best-fit parameters in water can also be found in Table 2 in main text, along with the nitrophenolates in basic water after 400 nm excitation for comparison.

[b] The fitted time zeros are notably larger in water (~0.4 ps) than those in methanol (~0.04 ps), visualized by the data plots following the common 267 nm excitation in Figure S4.

[c] The pertinent lifetimes in methanol are less accurately determined (likely shortened) versus water due to the pre-excitation spectral subtraction procedure described Figure S5.

The probe-dependent fits of three nitrophenolates in methanol and water data reveal clear differences in the early-time dynamics, mainly due to the early-time delay of prominent TA

features in water. After temporally adjusting the water data to account for this onset (i.e., by shifting the time axis by the time-zero offset, t_0, from the fitting procedure), the similarity between the late-time TA features in both solvents becomes apparent. Apart from the time-zero offset, the features are generally lengthened in methanol, particularly for the HGSA decay at late times, which is consistent with a longer solvation time of methanol than water [20,21].

Moreover, the late-time absorption band of nitrophenolates that appears in water after 267 nm excitation (seen as the trapped state in global analysis, see cyan traces in Figure S2d,e,f) does not appear in the methanol data (more evident in Figure S4). This finding could be due to the nonzero background signal in methanol (see Figure S5 above, and Section 2.3 in main text), hypothesized to arise from a long-lasting weak absorption band from some specific solute-solvent interactions involving methanol molecules (e.g., a long-lived trapped state that does not get effectively quenched by methanol [22], yet the counterpart in water completely diminishes within the laser pulse train interval of 1 ms). In order to accurately fit the nitrophenolate TA data across the detection time window with reliable lifetimes, spectral subtraction was performed in a consistent manner (Figure S5c,f), which unfortunately makes it challenging to characterize the real or potential trapped state in methanol. Future work with much longer detection time windows is necessary to shed more light on this specific point and what underlies this light-induced yet stagnant spectral feature.

Table S2. Parameters[a] for the probe-dependent fits of nitrophenols in water and methanol after 267 nm excitation (TA data plots shown in Figure S7 above).

Mol.	Solvent	Region center (nm)	t_0 (ps)	FWHM (ps)	τ_1 (ps)	τ_2 (ps)	τ_3 (ps)	τ_4 (ps)	τ_5 (ps)	τ_6 (ns)	τ_7 (ns)
2NP	Water	515	0.08	0.33	0.04	0.69	0.88	2000			
		615	0.07	0.39	0.06	0.14	9.1	960			
	Methanol	515	0	0.32	0.05	0.07	0.28	2.3	96		
		615	−0.02	0.39	0.04	0.1	0.16	2.4	190		
4NP	Water	515	−0.02	0.36	0.10	0.35	0.30	3.1	58	5.4	
		615	−0.02	0.36	0.09	0.31	0.20	1.8	23	9.7	
	Methanol	515	0	0.32	0.04	0.14	7.1	9.1	8.9	0.11	1.3
		615	0	0.28	0.03	0.19	7.5	9.1	12	0.081	2.5
3NP	Water	515	0.06	0.31	0.29	2.8	750				
		615	0.06	0.31	0.03	1.8	2000				
	Methanol	515	0.04	0.31	0.05	3.4	2.2	8.9	290		
		615	−0.03	0.31	0.15	9.8	1.2	285	84[b]		

[a] Values are reported to 2 significant figures (sig. fig.) or to the nearest 10 fs (whichever is more restrictive). The "Region center" column is the center wavelength in nm unit for the TA signal to be integrated and averaged for the least-squares fitting analysis. Most of the lifetimes are not as accurate as shown here, but are reported here exactly as found from the fitting for model transparency. Each probe-dependent data set, fit with a sequential model, required only four components to fit well. Explanations of the parameters can be found in the Methods (see Section 4.5 in main text). Blue and green shades highlight the retrieved parameters from fs-TA spectra of the (protonated) nitrophenol samples after 267 nm excitation in water and methanol, respectively. For comparison, the best-fit parameters in water and methanol using global analysis (see Figure S6 above) can also be found in Table 3 in main text.

[b] This time constant seems to fit the unusual intensity drop in a broad absorption band at later times for 3NP in methanol after 267 nm excitation (see green trace beyond ~100 ps in Figure S7f).

Notably, the probe-dependent fits of three nitrophenols in water and methanol show clear differences in late-time features post 267 nm excitation. In contrast to the nitrophenolates, the

ESA bands in both solvents rise at almost the exact same time (i.e., no temporal shift is required for a visual match, see Figure S4 for comparison), with a common fitted FWHM of ~300–400 fs that is consistent with the uncompressed 267 nm laser pump pulse duration (Section 4.3). However, the late-time features are generally very different (Figure S7) while they are quite similar for the nitrophenolates (Figure S4). Besides intrinsic differences between the electronic structures of nitrophenols and nitrophenolates [1], the ESIPT/ESPT capability of 2NP and ESPT capability of 4NP (3NP, to a lesser extent) in water likely cause them to exhibit different dynamics than those in methanol wherein the proton transfer processes are hindered [5,12].

One interesting point is that for 4NP in methanol at long-wavelength region (590–640 nm, Figure S7d), the small dip in ESA band around 200 fs (after the initial ESA) requires a small SE feature to fit, which emerges at almost the same time as the prominent SE in water (see blue trace in Figure S7d), in accord with 2D-contour plots showing an SE band in the long-wavelength region (Figure 6b,e in main text). This result implies that the SE band stems from ultrafast nitro-group twisting motions that can lead some of the molecules toward a TICT state (reminiscent of $2/4NP^-$ cases in Figure 5a), not concerning the other subpopulation that could undergo ES(I)PT in 2NP or ESPT in 4NP. The same pattern holds for 2NP in methanol (Figure S7b), although the SE band seems to appear earlier than that in water, corroborated by 2D-contour plots (Figure 6a,d in main text). In particular, the clearly delayed SE peak maximum in water for 2NP (see the orange line across Figure S7b,d) is in accord with the intramolecular H-bonding chain (see 2NP chemical structure in Figure 1 inset) that needs to break for the nitro twist to occur [14], whereas this delay is insignificant for 4NP in water versus methanol (Figure S7d) because the nitro group is at the *para* site to the phenolic hydroxy group. Such features cannot be readily retrieved from global analysis, likely because they are very weak and rather localized to the long-wavelength region (i.e., not visible or apparent below ~530 nm).

These correlating results suggest that some of the initial excited-state relaxation processes (e.g., a swift twist of the nitro group) are conserved in both solvents (water and methanol), despite the large differences in the overall dynamics on the ps-to-ns timescale. Further studies on solvent effects could help to determine the correlations between the initial PES (e.g., moving out of the Franck-Condon region) and the late-time PES (e.g., close to a conical intersection or a lower-lying electronic state that enables efficient nonradiative decay back to S_0) [5,14].

Table S3. Calculated ground state Raman and experimental FSRS peak frequencies with vibrational normal mode assignments of 2-nitrophenolate ($2NP^-$) in water.

Cal. freq. (cm^{-1})[a]	Cal. freq. (cm^{-1})[b]	Exp. freq. (cm^{-1})[c]	Mode assignment (major)[d]
1638	1614 (0.985)	1616	C=C and C=O str.
1560	1553 (0.995)	1556	C=O, C=C, and $-NO_2$ str.
1474	1452 (0.985)	1451	C=C str., H-rocking
**1329**	---	**1332**	**C–C str., CNO bend**
1237	---	1249	C–N str., H-rocking, H-sc.
1150	1139 (0.99)	1137	H-sc.
1083	1078 (0.995)	1079	C–N str., ring def.
1028	1023 (0.995)	1024	Ring def./breathing, H-motions
885	872 (0.985)	869	Ring def., C–N str., $-NO_2$ sc.
810	---	813	Ring breathing, $-NO_2$ sc.
656	---	656	Ring def., $-NO_2$ sc.
562	556 (0.99)	555	Ring def.
429	---	428	Ring def., $-NO_2$ bend, C=O rocking
381	---	380	Ring def.

[a] The unscaled ground state (GS) Raman peak frequencies were calculated using the DFT/B3LYP/6-311+G(d,p) as the method/functional/basis sets of the geometrically optimized structure in the electronic ground state (S_0).

[b] The calculated GS Raman peak frequencies were scaled to better match the experimental frequencies, the scaling factors for each individual peak are listed in parentheses with unscaled frequencies left blank.

[c] The experimental GS-FSRS peak frequencies were determined by fitting the experimental spectrum of $2NP^-$ in basic water with a least-squares-fit gaussian profile, and the center frequency was extracted.

[d] The abbreviations for major vibrational motions are: stretching (str.), scissoring (sc.), ring deformation (ring def.), and hydrogen motions (H-motions). The strongest peak within the spectral range is bolded (see Figure S9a, red trace).

Table S4. Calculated ground state Raman and experimental FSRS peak frequencies with vibrational normal mode assignments of 3-nitrophenolate ($3NP^{-}$) in water.

Cal. freq. (cm^{-1})[a]	Cal. freq. (cm^{-1})[b]	Exp. freq. (cm^{-1})[c]	Mode assignment (major)[d]
1615	---	1614	C=C and C=O str.
1550	---	1558	C=C str.
1494	---	1499	C=O str., $–NO_2$ str., H-rocking
**1324**	---	**1350**	**C–N str., $–NO_2$ sc.**
1304	1278 (0.98)	1273	H-rocking, ring def.
1076	---	1078	C–N str., ring def., H-sc.
991	---	987	Ring def., weak C–N str.
830	817 (0.985)	815	Ring breathing, $–NO_2$ sc.
562	---	569	Ring def., $–NO_2$ rocking, H-rocking
527	---	531	Ring def.

[a] The unscaled ground state (GS) Raman peak frequencies were calculated using the DFT/B3LYP/6-311+G(d,p) as the method/functional/basis sets of the geometrically optimized structure in the electronic ground state (S_0).

[b] The calculated GS Raman peak frequencies were scaled to better match the experimental frequencies, the scaling factors for each individual peak are listed in parentheses with unscaled frequencies left blank.

[c] The experimental GS-FSRS peak frequencies were determined by fitting the experimental spectrum of $3NP^{-}$ in basic water with a least-squares-fit gaussian profile, and the center frequency was extracted.

[d] The abbreviations for major vibrational motions are: stretching (str.), scissoring (sc.), ring deformation (ring def.), and hydrogen motions (H-motions). The strongest peak within the spectral range is bolded (see Figure S9b, red trace).

Table S5. Calculated ground state Raman and experimental FSRS peak frequencies with vibrational normal mode assignments of 4-nitrophenolate ($4NP^-$) in water.

Cal. freq. (cm^{-1})[a]	Cal. freq. (cm^{-1})[b]	Exp. freq. (cm^{-1})[c]	Mode assignment[d]
1622	1581 (0.975)	1585	C=C str.
1549	1533 (0.99)	1534	C=O str., C=C str., C–N str.
1508	1477 (0.98)	1478	Asym. C=C str.
1321	---	1337	H-rocking, weak C=C str.
**1275**	---	**1287**	**C–N str., –NO_2 str., H-sc.**
1272	1240 (0.975)	1236	H-rocking, weak C–C str.
1179	1167 (0.99)	1168	H-sc., –NO_2 str.
1128	1111 (0.985)	1114	H-sc., C–N str.
841	---	848	Ring breathing, –NO_2 sc.
813	---	813	Ring def., –NO_2 sc.
631	628 (0.995)	627	Ring def.
547	---	585	–NO_2 rocking, H-rocking
446	---	448	–NO_2, C=O, and H rocking
368	---	371	Ring def.

[a] The unscaled ground state (GS) Raman peak frequencies were calculated using the DFT/B3LYP/6-311+G(d,p) as the method/functional/basis sets of the geometrically optimized structure in the electronic ground state (S_0).

[b] The calculated GS Raman peak frequencies were scaled to better match the experimental frequencies, the scaling factors for each individual peak are listed in parentheses with unscaled frequencies left blank.

[c] The experimental GS-FSRS peak frequencies were determined by fitting the experimental spectrum of $4NP^-$ in basic water with a least-squares-fit gaussian profile, and the center frequency was extracted.

[d] The abbreviations for major vibrational motions are: stretching (str.), scissoring (sc.), ring deformation (ring def.), and hydrogen motions (H-motions). The strongest peak within the spectral range is bolded (see Figure S9c, red trace).

Table S6. Calculated ground state Raman and experimental FSRS peak frequencies with vibrational normal mode assignments of 2-nitrophenol (2NP) in water.

Cal. freq. (cm^{-1})[a]	Cal. freq. (cm^{-1})[b]	Exp. freq. (cm^{-1})[c]	Mode assignment (major)[d]
1613	1596 (0.99)	1597	C=C str., H-rocking
1552	1529 (0.985)	1532	$-NO_2$ str., C=C str., H-rocking
1466	1459 (0.995)	1462	$-NO_2$ str., H-rocking, C=C str.
**1404**	**1375 (0.98)**	**1357**	**H-rocking, C=C str., $-NO_2$ str.**
1302	---	1309	C–N str., $-NO_2$ str., H-rocking
1261	1254 (0.995)	1253	CO str., $-NO_2$ str., C–N str.
1156	1138 (0.985)	1140	H-sc., C–N str.
1047	1030 (0.985)	1032	Ring breathing, H-rocking, H-sc.
881	873 (0.99)	875	Ring def., $-NO_2$ sc., C–N str., CO str.
833	825 (0.99)	818	Ring breathing, $-NO_2$ sc., CO str.
569	564 (0.99)	566	Ring def., $-NO_2$ rocking
423	419 (0.99)	420	$-NO_2$ rocking, $-NO_2$ and COH sc.
288	---	288	$-NO_2$ and COH rocking

[a] The unscaled ground state (GS) Raman peak frequencies were calculated using the DFT/B3LYP/6-311+G(d,p) as the method/functional/basis sets of the geometrically optimized structure in the electronic ground state (S_0).

[b] The calculated GS Raman peak frequencies were scaled to better match the experimental frequencies, the scaling factors for each individual peak are listed in parentheses with unscaled frequencies left blank.

[c] The experimental GS-FSRS peak frequencies were determined by fitting the experimental spectrum of 2NP in acidic water with a least-squares-fit gaussian profile, and the center frequency was extracted. These frequencies could differ by 1–2 cm^{-1} from the apparent spectral peak locations.

[d] The abbreviations for major vibrational motions are: stretching (str.), scissoring (sc.), ring deformation (ring def.), and hydrogen motions (H-motions). The strongest peak within the spectral range is bolded (see Figure S10a, red trace).

Table S7. Calculated ground state Raman and experimental FSRS peak frequencies with vibrational normal mode assignments of 3-nitrophenol (3NP) in water.

Cal. freq. (cm^{-1})[a]	Cal. freq. (cm^{-1})[b]	Exp. freq. (cm^{-1})[c]	Mode assignment (major)[d]
1621	1605 (0.99)	1604	C=C str.
**1353**	**---**	**1359**	**C–N str., –NO_2 str.**
1335	1315 (0.985)	1315	H-rocking, H-sc.
1267	---	1263	CO str., H-rocking, C–N str.
1097	1075 (0.98)	1075	Ring def., H-rocking, H-sc., C–N str.
1010	995 (0.985)	1001	Ring def.
827	811 (0.98)	802	Ring def., –NO_2 sc., CO str.
562	---	587	Ring def., –NO_2 rocking
517	507 (0.98)	487	Ring def.
307	302 (0.985)	298	HOOP

[a] The unscaled ground state (GS) Raman peak frequencies were calculated using the DFT/B3LYP/6-311+G(d,p) as the method/functional/basis sets of the geometrically optimized structure in the electronic ground state (S_0).

[b] The calculated GS Raman peak frequencies were scaled to better match the experimental frequencies, the scaling factors for each individual peak are listed in parentheses with unscaled frequencies left blank.

[c] The experimental GS-FSRS peak frequencies were determined by fitting the experimental spectrum of 3NP in acidic water with a least-squares-fit gaussian profile, and the center frequency was extracted.

[d] The abbreviations for major vibrational motions are: stretching (str.), scissoring (sc.), ring deformation (ring def.), hydrogen motions (H-motions), and hydrogen out-of-plane (HOOP) motions. The strongest peak within the spectral range is bolded (see Figure S10b, red trace).

Table S8. Calculated ground state Raman and experimental FSRS peak frequencies with vibrational normal mode assignments of 4-nitrophenol (4NP) in water.

Cal. freq. (cm^{-1})[a]	Cal. freq. (cm^{-1})[b]	Exp. freq. (cm^{-1})[c]	Mode assignment (major)[d]
1627	1602 (0.985)	1603	C=C str.
1524	1509 (0.99)	1514	$-NO_2$ str., C=C str., H-sc.
1462	---	1463	C=C str., $-NO_2$ str., H-sc.
**1336**	**---**	**1341**	**$-NO_2$ str., C–N str., CO str.**
1289	---	1288	CO str., C–N str., H-rocking
1120	1114 (0.995)	1112	H-sc., C–N str.
875	866 (0.99)	863	Ring breathing, $-NO_2$ sc.
637	618 (0.97)	585	Ring def., $-NO_2$ sc.
495	480 (0.97)	456	HOOP
420	---	417	HOOP
293	---	305	HOOP

[a] The unscaled ground state (GS) Raman peak frequencies were calculated using the DFT/B3LYP/6-311+G(d,p) as the method/functional/basis sets of the geometrically optimized structure in the electronic ground state (S_0).

[b] The calculated GS Raman peak frequencies were scaled to better match the experimental frequencies, the scaling factors for each individual peak are listed in parentheses with unscaled frequencies left blank.

[c] The experimental GS-FSRS peak frequencies were determined by fitting the experimental spectrum of 4NP in acidic water with a least-squares-fit gaussian profile, and the center frequency was extracted.

[d] The abbreviations for major vibrational motions are: stretching (str.), scissoring (sc.), ring deformation (ring def.), hydrogen motions (H-motions), and hydrogen out-of-plane (HOOP) motions. The strongest peak within the spectral range is bolded (see Figure S10c, red trace).

3. Supplementary References

1. Wanko, M.; Houmøller, J.; Støchkel, K.; Suhr Kirketerp, M.-B.; Petersen, M.Å.; Nielsen, M.B.; Nielsen, S.B.; Rubio, A. Substitution effects on the absorption spectra of nitrophenolate isomers. *Phys. Chem. Chem. Phys.* **2012**, *14*, 12905–12911.
2. Liu, S. Where does the electron go? The nature of *ortho*/*para* and *meta* group directing in electrophilic aromatic substitution. *J. Chem. Phys.* **2014**, *141*, 194109.
3. van Stokkum, I.H.M.; Larsen, D.S.; van Grondelle, R. Global and target analysis of time-resolved spectra. *Biochim. Biophys. Acta* **2004**, *1657*, 82–104.
4. Snellenburg, J.J.; Laptenok, S.P.; Seger, R.; Mullen, K.M.; van Stokkum, I.H.M. Glotaran: A Java-based graphical user interface for the R-package TIMP. *J. Stat. Softw.* **2012**, *49*, 1–22.
5. Fang, C.; Tang, L.; Chen, C. Unveiling coupled electronic and vibrational motions of chromophores in condensed phases. *J. Chem. Phys.* **2019**, *151*, 200901.
6. Chen, C.; Tutol, J.N.; Tang, L.; Zhu, L.; Ong, W.S.Y.; Dodani, S.; Fang, C. Excitation ratiometric chloride sensing in a standalone yellow fluorescent protein is powered by the interplay between proton transfer and conformational reorganization. *Chem. Sci.* **2021**, *12*, 11382–11393.
7. Krueger, T.D.; Tang, L.; Giesbers, G.; Van Court, R.C.; Zhu, L.; Robinson, S.C.; Ostroverkhova, O.; Fang, C. Ultrafast triplet state formation in a methylated fungi-derived pigment: Toward rational molecular design for sustainable optoelectronics. *J. Phys. Chem. C* **2021**, *125*, 17565–17572.
8. Schweigert, N.; Zehnder, A.J.B.; Eggen, R.I.L. Chemical properties of catechols and their molecular modes of toxic action in cells, from microorganisms to mammals. *Environ. Microbiol.* **2001**, *3*, 81–91.
9. Romero, R.; Salgado, P.R.; Soto, C.; Contreras, D.; Melin, V. An experimental validated computational method for pK_a determination of substituted 1,2-dihydroxybenzenes. *Front. Chem.* **2018**, *6*, 208.
10. Turner, M.A.P.; Turner, R.J.; Horbury, M.D.; Hine, N.D.M.; Stavros, V.G. Examining solvent effects on the ultrafast dynamics of catechol. *J. Chem. Phys.* **2019**, *151*, 084305.
11. Wang, Y.; Liu, W.; Tang, L.; Oscar, B.G.; Han, F.; Fang, C. Early time excited-state structural evolution of pyranine in methanol revealed by femtosecond stimulated Raman spectroscopy. *J. Phys. Chem. A* **2013**, *117*, 6024–6042.
12. Simkovitch, R.; Shomer, S.; Gepshtein, R.; Huppert, D. How fast can a proton-transfer reaction be beyond the solvent-control limit? *J. Phys. Chem. B* **2015**, *119*, 2253–2262.
13. Sasaki, S.; Drummen, G.P.C.; Konishi, G.-i. Recent advances in twisted intramolecular charge transfer (TICT) fluorescence and related phenomena in materials chemistry. *J. Mater. Chem. C* **2016**, *4*, 2731–2743.
14. Kumpulainen, T.; Lang, B.; Rosspeintner, A.; Vauthey, E. Ultrafast elementary photochemical processes of organic molecules in liquid solution. *Chem. Rev.* **2017**, *117*, 10826–10939.
15. Tang, L.; Fang, C. Nitration of tyrosine channels photoenergy through a conical intersection in water. *J. Phys. Chem. B* **2019**, *123*, 4915–4928.
16. He, X.; Bell, A.F.; Tonge, P.J. Ground state isomerization of a model green fluorescent

protein chromophore. *FEBS Lett.* **2003**, *549*, 35–38.
17. Xu, C.; Yu, L.; Zhu, C.; Yu, J.; Cao, Z. Intersystem crossing-branched excited-state intramolecular proton transfer for o-nitrophenol: An ab initio on-the-fly nonadiabatic molecular dynamic simulation. *Sci. Rep.* **2016**, *6*, 26768.
18. Boulanger, S.A.; Chen, C.; Myasnyanko, I.N.; Baranov, M.S.; Fang, C. Fluorescence modulation of *ortho*-green fluorescent protein chromophores following ultrafast proton transfer in solution. *J. Phys. Chem. B* **2022**, *126*, 5081–5093.
19. Nibbering, E.T.J.; Fidder, H.; Pines, E. Ultrafast chemistry: Using time-resolved vibrational spectroscopy for interrogation of structural dynamics. *Annu. Rev. Phys. Chem.* **2005**, *56*, 337–367.
20. Agmon, N.; Huppert, D.; Masad, A.; Pines, E. Excited-state proton-transfer to methanol water mixtures. *J. Phys. Chem.* **1991**, *95*, 10407–10413.
21. Horng, M.L.; Gardecki, J.A.; Papazyan, A.; Maroncelli, M. Subpicosecond measurements of polar solvation dynamics: Coumarin 153 revisited. *J. Phys. Chem.* **1995**, *99*, 17311–17337.
22. Maillard, J.; Klehs, K.; Rumble, C.; Vauthey, E.; Heilemann, M.; Fürstenberg, A. Universal quenching of common fluorescent probes by water and alcohols. *Chem. Sci.* **2021**, *12*, 1352–1362.
